# [3-^11^C]Pyruvate PET detects alterations in cardiac pyruvate metabolism induced by doxorubicin chemotherapy

**DOI:** 10.1038/s44303-026-00165-8

**Published:** 2026-04-16

**Authors:** Chul-Hee Lee, Thomas Ruan, Shuvra Debnath, Anja S. Wacker, Grace Figlioli, John W. Babich, Sadek A. Nehmeh, Kayvan R. Keshari, James M. Kelly

**Affiliations:** 1 Molecular Imaging Innovations Institute (MI3), Weill Cornell Medicine, New York, NY USA; 2 Department of Radiology, Weill Cornell Medicine, New York, NY USA; 3 Department of Radiology and Molecular Pharmacology Program, Memorial Sloan Kettering Cancer Center, New York, NY USA; 4 Citigroup Biomedical Imaging Center, Weill Cornell Medicine, New York, NY USA; 5 Sandra and Edward Meyer Cancer Center, Weill Cornell Medicine, New York, NY USA; 6 Present Address: Ratio Therapeutics, Boston, MA USA

**Keywords:** Cardiology, Physiology

## Abstract

Changes in cardiac metabolism typically precede cardiac dysfunction and therefore represent an important target for diagnosis and treatment designed to prevent progression to heart failure, a leading cause of death. Profound changes in pyruvate metabolism, including reduced expression of the mitochondrial pyruvate carrier (MPC), are increasingly recognized as early maladaptive alterations in cardiomyopathies, but no methods currently exist to determine MPC expression in vivo. We exposed mice to doxorubicin (DOX), an anthracycline chemotherapeutic known to perturb pyruvate metabolism, and demonstrated that cardiac tissue levels of MPC decrease within 4 weeks of initial DOX exposure. Using a combination of stable isotope tracing metabolomics, hyperpolarized [1-^13^C]pyruvate magnetic resonance imaging (MRI), and [3-^11^C]pyruvate positron emission tomography (PET), we found that loss of MPC and monocarboxylate transporter 1 (MCT1) resulted in decreased utilization of pyruvate for mitochondrial oxidative metabolism and resulted in decreased cardiac carbon-11 clearance. Despite recovery of expression levels of pyruvate transporters, including MPC, 16 weeks after initial DOX exposure, cardiac carbon-11 clearance still trends towards differences between control mice and the mice exposed to this chemotherapeutic. [3-^11^C]Pyruvate PET is therefore a promising approach to imaging cardiac pyruvate transport with potential applications to the identification of early maladaptive changes in pyruvate metabolism and monitoring response to therapy.

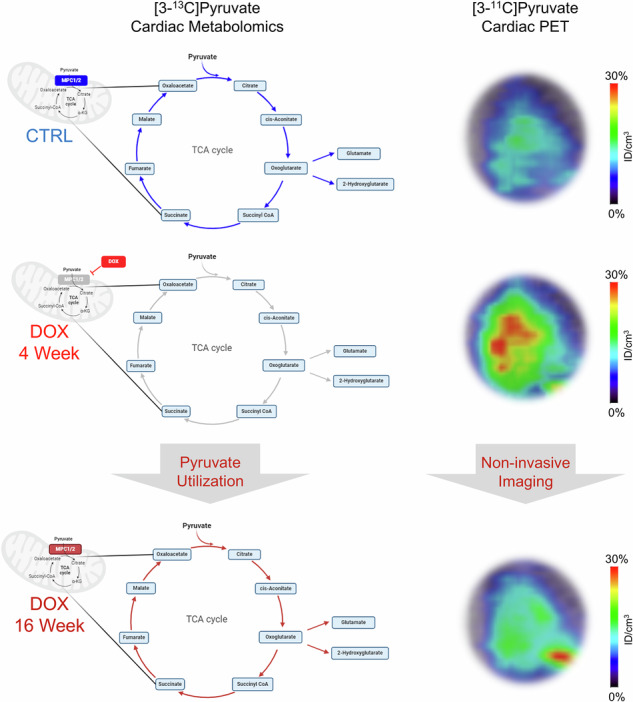

## Introduction

Heart disease is a leading cause of death worldwide^1^. Although the etiology of heart failure is diverse, metabolic perturbation is a common feature. Given the prevalence of this condition, methods of imaging cardiac metabolism could play a critical role in the treatment of millions of patients. Significantly, metabolic dysfunction precedes functional and structural changes, thereby identifying these processes as diagnostic and therapeutic targets of great potential significance. In this context, molecular and cellular imaging using probes targeting cardiac metabolism potentially offers a powerful approach to advancing precision medicine in cardiology^2^.

Heart failure may present with reduced ejection fraction (HFrEF) or preserved ejection fraction (HFpEF). One important subset of HFrEF is anthracycline cardiotoxicity. Anthracycline chemotherapeutics (e.g., doxorubicin, DOX) are widely used and effective against numerous cancers^3^. However, their use is compromised by the development of cardiotoxicity. This condition may present as acute, early (within 1 year of treatment), or late onset (several years after treatment). There are no established strategies for distinguishing between reversible and progressive cardiac dysfunction^4^. Furthermore, predicting late-onset disease is challenging due to the combination of treatments that patients may receive and the lack of biomarkers of cardiac damage that precede dysfunction. These observations highlight the critical need to develop methods of early detection of anthracycline-induced cardiac injury that are independent of assessments of cardiac structure and function to enable timely intervention that may prevent irreversible cardiac damage and long-term heart failure. Such methods may also prove valuable in HFrEF of other etiology by enabling earlier detection of dysfunctional mitochondrial metabolism and implementation of disease-ameliorating treatment.

The critical role of metabolic reprogramming in promoting cardiac functional and structural changes after DOX exposure is increasingly recognized^5–7^, but there remains a lack of extensive longitudinal studies examining the key molecular mediators that regulate these adaptations. Mitochondria are known targets of DOX-induced toxicity in cardiomyocytes. These cells have the highest mitochondria content of any cell type^8^, highlighting the value of targeting biochemical processes that occur in mitochondria as a means of imaging pathological cardiac metabolic reprogramming. Under normal conditions, the heart derives more than 95% of its ATP from oxidative phosphorylation (OXPHOS)^9,10^, which relies on oxidation of substrates in the tricarboxylic acid (TCA) cycle. In HFrEF, glucose consumption by the TCA cycle decreases as glycolysis becomes uncoupled from OXPHOS^10,11^. Pyruvate is a key metabolite that links glycolysis to mitochondrial oxidative metabolism. It also lies at the nexus of other metabolic processes, including reduction to lactate, transamination to alanine, and conversion to oxaloacetate by pyruvate carboxylase. The potential for using this metabolite to image physiological cardiac metabolism and disease-related changes has been demonstrated by hyperpolarized (HP) [1-^13^C]pyruvate magnetic resonance imaging (MRI) in both animals and human subjects^12–16^. Despite the encouraging developments in HP [1-^13^C]pyruvate cardiac MRI, the short half-life of the hyperpolarized species (T1 relaxation time = 1 min) and the requirement for supra-physiological masses of pyruvate may ultimately limit some applications of this technology for cardiac imaging^17^. In this context, it is notable that ^11^C-labeled pyruvate positron emission tomography (PET) has not been widely pursued as a complementary or alternative imaging strategy. The physical properties of carbon-11 (*t*_1/2_ = 20.4 min), which decays almost entirely by positron emission (*β*^+^ = 99.8%, *E*_avg_ = 385.7 keV), render ^11^C-labeled pyruvate an intriguing candidate for imaging changes in cardiac pyruvate metabolism. Two isotopologues of ^11^C-labeled pyruvate, [1-^11^C]pyruvate and [3-^11^C]pyruvate, are available in sufficient activities and purities for PET imaging^18–20^. Both isotopologues can be converted to ^11^C-labeled lactate, alanine, or glutamate. However, when imported into mitochondria, [1-^11^C]pyruvate is rapidly metabolized to acetyl-CoA and [^11^C]CO_2_, resulting in minimal retention of radioactivity in tissue^19^. By contrast, the metabolism of [3-^11^C]pyruvate does not produce [^11^C]CO_2_ so rapidly, resulting in longer cardiac retention and, potentially, a superior ability to assess changes in pyruvate metabolism.

Pyruvate requires monocarboxylate transporters (MCTs) for transport into cells and the mitochondrial pyruvate carriers 1 and 2 (MPC1/2) for transport across the mitochondrial membrane^21,22^. Since the discovery of MPC1/2 as essential transporters for pyruvate across the mitochondrial membrane, their role in cardiac metabolic changes associated with heart failure has been increasingly recognized^23–27^. Inhibition of pyruvate transport by DOX in rat cardiomyocytes was previously reported^28^, suggesting that MPC1/2 might contribute to DOX-induced metabolic disruption in cardiomyocytes. In this study, we hypothesize that DOX exposure compromises murine cardiac pyruvate metabolism by disrupting its transport and reducing expression of MCT and MPC1/2. We further hypothesize that DOX-induced changes in pyruvate metabolism can be detected and quantified through dynamic cardiac PET imaging using [3-^11^C]pyruvate. This study uses stable isotope tracing and HP [1-^13^C]pyruvate MRI to support the conclusion that clearance of radioactivity from cardiac tissue following administration of [3-^11^C]pyruvate corresponds with pyruvate transport and utilization by the TCA cycle.

## Results

### MPC1/2 expression decreases in the murine heart after exposure to doxorubicin

**Fig. 1:**
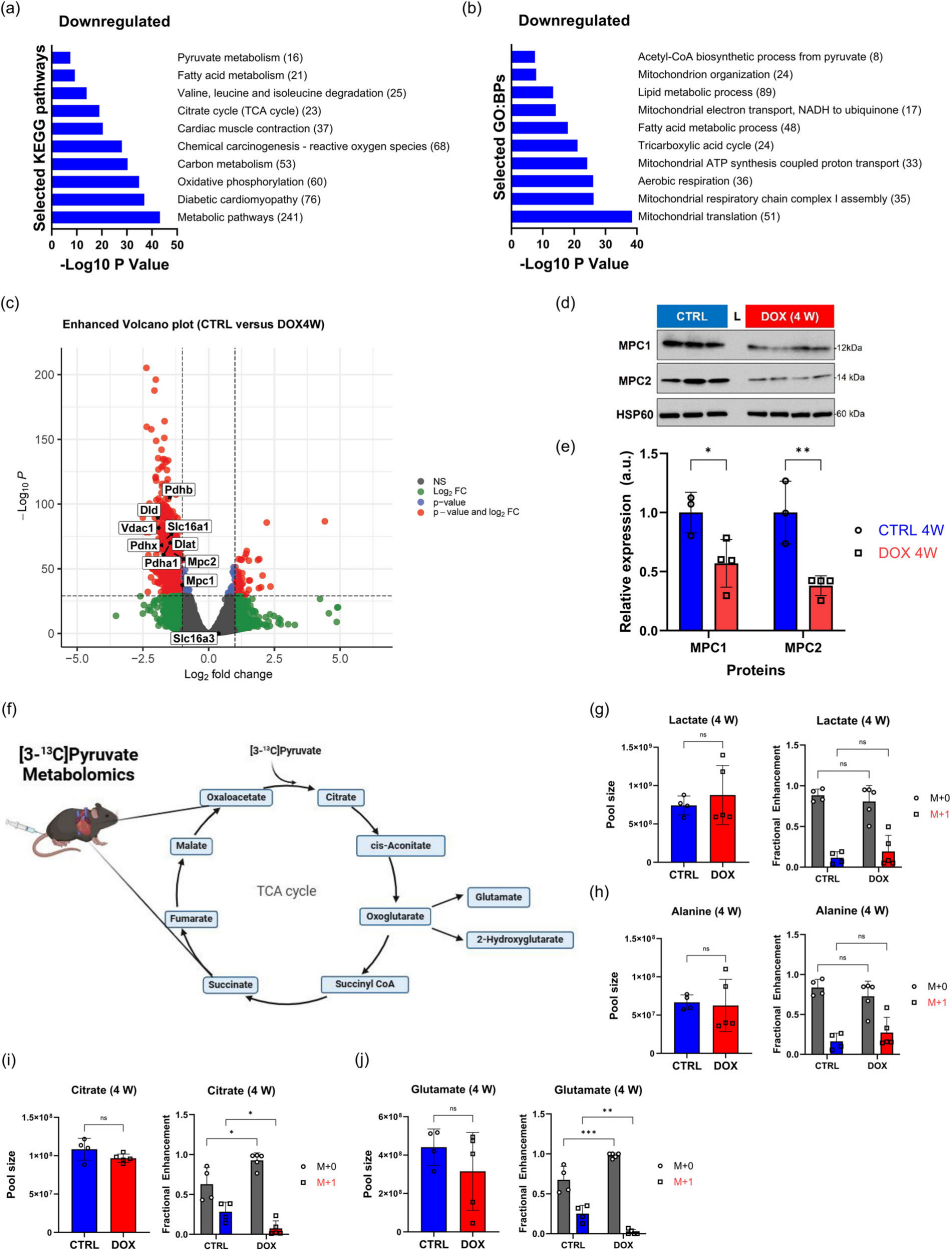
DOX exposure leads to decreased cardiac pyruvate metabolism in mice. **a** Selected significantly downregulated KEGG pathways at 4 weeks after DOX exposure. **b** Selected significantly downregulated GO:BPs at 4 weeks after DOX exposure. The number of genes in each pathway is indicated in parenthesis. **c** EnhancedVolcano plot from the bulk RNA sequencing performed on cardiac tissue samples collected at 4 weeks (Log2 fold change (FC) > | 1 | , *p* < 0.0001). Selected genes associated with pyruvate transport and metabolism, Mpc1, Mpc2, Pdhb, Pdha1, Dlat, Dld, Vdac1, Pdhx, Slc16a1 (monocarboxylate transporter 1), and Slc16a3 (monocarboxylate transporter 4) are highlighted. **d** Western blot analysis of cardiac MPC1 and MPC2 expression. HSP60 was used as a reference. **e** ROI quantification of each protein expression level was performed using ImageJ. **f** Map of TCA cycle intermediates assessed during [3-^13^C]pyruvate stable isotope tracing metabolomics experiments. The illustration was created with BioRender. **g–j** Comparison of total pool size and M + 0 and M + 1 fractional enrichments of lactate (**g**), alanine (**h**), citrate (**i**), and glutamate (**j**) between the control and DOX groups in hearts collected 10 min p.i. of [3-^13^C]pyruvate. The experiment was performed 4 weeks after the first DOX exposure. Data are presented as the mean ± s.d. *p < 0.05; **p < 0.01, ***p < 0.001. Statistical analysis was performed using two-way ANOVA (**e**, **g**–**j**). DOX doxorubicin, CTRL control, MPC mitochondrial pyruvate carrier, GO:BP gene ontology:biological process, KEGG Kyoto Encyclopedia of Genes and Genomes, TCA tricarboxylic acid, HSP60 heat shock protein 60, ROI Region of interest.

To assess the initial effect of DOX exposure on cardiac metabolism, we analyzed cardiac tissue samples collected from mice 4 weeks after the first administration of DOX. At this point, we previously observed cardiac atrophy in the mice exposed to DOX^29^, a finding that we confirmed in this study (Supplementary Fig. 1a). We performed bulk RNA sequencing analysis on these tissues and identified 1618 differentially expressed genes (DEGs) between the DOX and control groups (*p* < 0.0001, Log2 fold change (FC) > | 1 |). The DEGs were used for Gene Ontology (GO) and Kyoto Encyclopedia of Genes and Genomes (KEGG) pathway enrichment using the DAVID and STRING databases. Among the most significantly downregulated pathways were those involving pyruvate metabolism and processes supporting mitochondrial function (Fig. 1a, b and Supplementary Tables 1, 2). These findings are consistent with prior observations of mitochondrial oxidative stress induced by DOX^30^. From the EnhancedVolcano plot of the DEGs, we identified 9 significantly downregulated genes associated with pyruvate transport (Fig. 1c). The most significantly affected genes were not related to pyruvate metabolism (Supplementary Table 3). Two of the 9 genes, Mpc1 and Mpc2, are highly expressed in the hearts of mice and humans (Supplementary Fig. 1b, c and Supplementary Table 4) and encode subunits of the mitochondrial pyruvate carrier (MPC), which assembles as a heterodimer to facilitate pyruvate import into the mitochondrial matrix^21,22^. Given prior reports of MPC expression deficits in other cardiac pathologies^23–25,27^ and the interaction between MPC1/2 and the pyruvate dehydrogenase complex, which catalyzes the irreversible conversion of pyruvate to acetyl-CoA (Supplementary Fig. 1d and Supplementary Table 5), we evaluated the protein expression levels of MPC1 and MPC2 in the heart tissue samples. There was a significant reduction in MPC1 and MPC2 expression in the tissue taken from the mice exposed to DOX compared to the age-matched controls (*p* = 0.0248 for MPC1; *p* = 0.0028 for MPC2; Fig. 1d, e). In parallel, we observed a significant reduction in MCT1 transcripts (Slc16a1) and expression in these tissues (*p* = 0.0300; Fig. 1c and Supplementary Fig. 1e). By contrast, expression of MCT4 (Slc16a3) was not significantly different in the mice exposed to DOX compared to controls.

Next, we evaluated myocardial carbohydrate metabolism and tricarboxylic acid (TCA) cycle flux by stable isotope tracing in mice using [3-^13^C]pyruvate at the 4-week time point (Fig. 1f). Cardiac tissue was collected 10 min post-injection (p.i.). We chose this time point for the experiment because prior studies in healthy mouse hearts indicated that incorporation of multiple isotopic labels into TCA cycle intermediates, corresponding to multiple iterations of the TCA cycle, was evident in samples collected 10 min p.i. (Supplementary Fig. 1f). These experiments revealed no significant changes in the total lactate, alanine, citrate, and glutamate pools in hearts collected from the animals exposed to DOX at 10 min p.i. compared to controls (Fig. 1g–j), although the total cardiac metabolite pool size was significantly lower (*p* = 0.0079) in the mice exposed to DOX (Supplementary Fig. 1g). Fractional enrichment of the M + 1 peak in lactate was slightly increased in the hearts from the DOX animals (*n* = 5) compared to the controls (0.19 vs. 0.12; Fig. 1g), although we observed considerable variability within this group which prevented the difference from reaching statistical significance. We additionally observed reduced expression of lactate dehydrogenase (LDH) A and B, while there was no change in alanine aminotransferase (ALT) at the 4-week time point in mice exposed to DOX (Supplementary Fig. 1e). In agreement with the lactate data, M + 1 fractional enrichment of alanine increased in the hearts exposed to DOX, although the variability within this group ensured that the difference to the control hearts was not statistically significant (Fig. 1h). By contrast, M + 1 fractional enrichment of citrate (*p* = 0.0230) and glutamate (*p* = 0.0052) was significantly lower in the hearts extracted from the mice exposed to DOX (Fig. 1i, j). Similarly, M + 1 fractional enrichment of other TCA cycle intermediates, including aconitate, fumarate, malate, oxoglutarate, and succinate, was significantly lower in the DOX hearts (Supplementary Fig. 1h). The pool size of these TCA cycle metabolites was generally comparable between the two groups, with only deficits in cardiac fumarate and malate in the DOX group reaching statistical significance. Given the reduced MPC1 and MPC2 expression in DOX-treated hearts and the corresponding decrease in M + 1 enrichment of TCA cycle metabolites, our findings suggest that DOX exposure impairs pyruvate entry into mitochondria and promotes its conversion to lactate and alanine in the cytosol.

### MPC1 and MPC2 are key mediators of pyruvate metabolism in human cardiomyocytes exposed to DOX

**Fig. 2:**
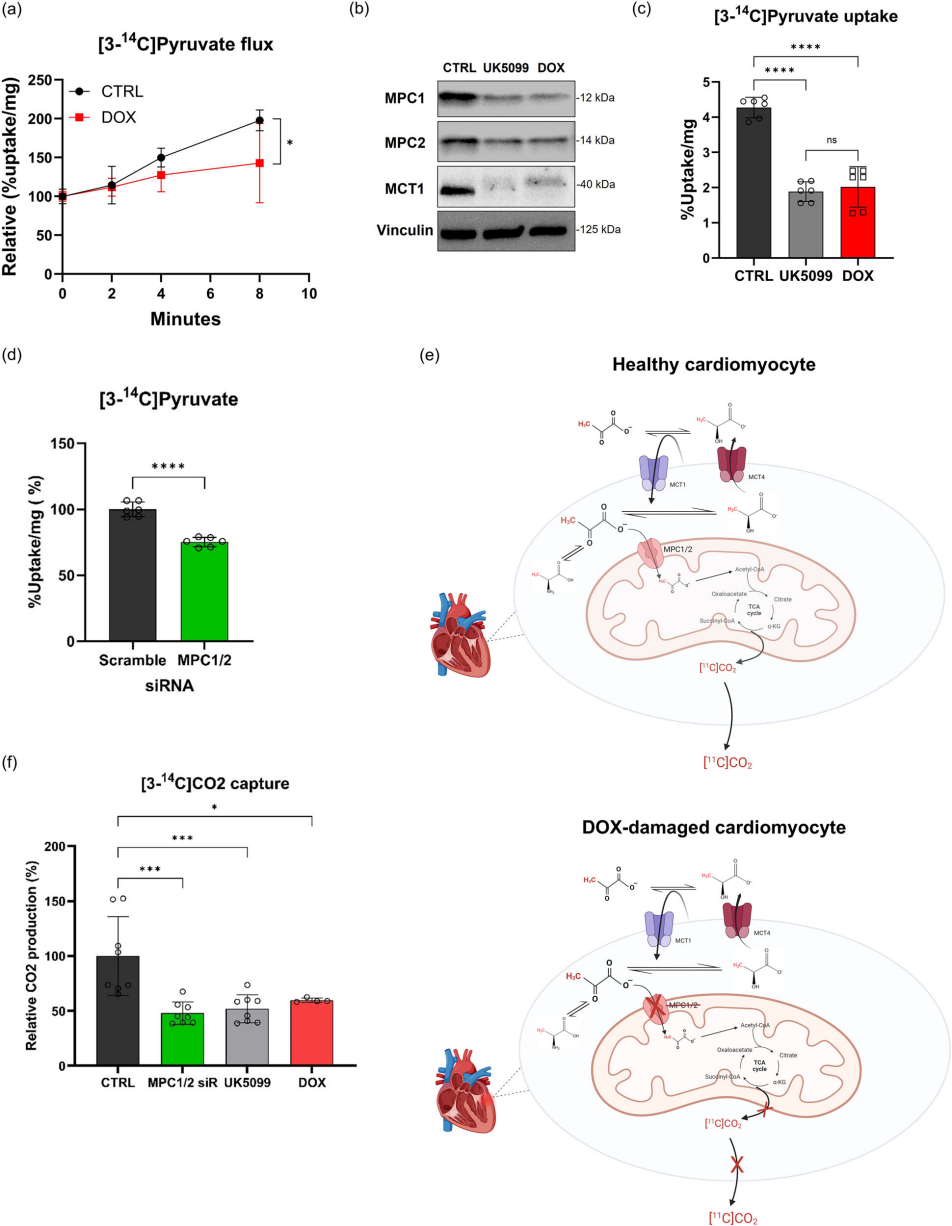
DOX exposure affects pyruvate uptake by reducing MPC1/2 in HCM. **a** [3-^14^C]Pyruvate flux in HCMs was evaluated in a time-dependent manner (0, 2, 4, 6, and 8 min). Cells were treated with vehicle (control; CTRL) or 0.1 µM DOX for 2 × 48 h (*n* = 6 per condition). **b** Western blot analysis of MPC1, MPC2, and MCT1 expression from HCM treated with vehicle (CTRL), UK5099 (100 µM for 72 h), or DOX (0.1 µM for 2 × 48 h). Vinculin was used as a reference. **c** [3-^14^C]Pyruvate uptake was assessed in HCMs treated with vehicle (CTRL), UK5099 (100 µM for 72 h), or DOX (0.1 µM for 2 × 48 h) for 8 min. **d** [3-^14^C]Pyruvate uptake in HCM treated with scramble siRNA (100 nM for 24 h) or MPC1/2 siRNA (50 nM of each MPC siRNA—total concentration of 100 nM—for 24 h) for 8 min. **e** Hypothesized effect of DOX treatment on the cellular fate of [3-^14^C]pyruvate, which can be retained within the cell as [3-^14^C]pyruvate or another labeled metabolite, released into the media as [3-^14^C]lactate, [3-^14^C]pyruvate, or another species, or released to the air as [^14^C]CO_2_. The illustration was created with BioRender. **f** [^14^C]CO₂ capture assay was performed following an 8-min incubation of HCM with [3-^14^C]pyruvate. Data are presented as the mean ± s.d. *p < 0.05; ***p < 0.001; ****p < 0.0001. Statistical analysis was performed using Pearson correlation coefficients (**a** *R*^2^ = 0.9728), two-way ANOVA (**c**), unpaired t-test (**d**), or one-way ANOVA (**f**). HCM human cardiomyocytes.

To test our hypothesis that DOX impairs mitochondrial pyruvate oxidation in part by targeting MPC1/2, we investigated whether DOX contributes to alterations in MPC1/2-mediated pyruvate metabolism in human cardiomyocytes. Consistent with previously established conditions^29,31^, human cardiomyocytes (HCM) were treated with 0.1 µM DOX for two consecutive 48-h periods (Supplementary Fig. 2a). There was no difference in cell viability between groups (Supplementary Fig. 2b,c). We incubated control and DOX-treated HCM with [3-^14^C]pyruvate for up to 8 min and observed significantly decreased time-dependent retention of signal in the DOX-treated HCMs (*p* = 0.0137; Fig. 2a). The difference in uptake in the DOX-treated HCMs was then compared to HCM treated with UK5099, a selective pharmacological inhibitor of MPC^32^, for 72 h before the addition of [3-^14^C]pyruvate, as shown in Supplementary Fig. 2a. These conditions were previously demonstrated to downregulate MPC1/2^33^. We confirmed downregulation of MPC1/2 in the UK5099- and DOX-treated cells and also determined by Western blotting that these treatments decreased expression of MCT1 (Fig. 2b). These experiments revealed that [3-^14^C]pyruvate uptake after 8 min was reduced by 2.3-fold in UK5099-treated HCM compared to the untreated control cells (*p* < 0.0001 for CTRL versus UK5099 or DOX; Fig. 2c). By contrast, [3-^14^C]pyruvate uptake was comparable between the UK5099-treated HCM and the DOX-treated HCM. These results indicate that DOX-induced changes in pyruvate metabolism are comparable to those induced by UK5099. Treatment with DOX or UK5099 did not affect cell viability but did result in a lower pH (6.8 vs. 7.5) in the media (Supplementary Fig. 2 d). We attributed this acidification to increased lactate production resulting from impaired mitochondrial import of pyruvate. In support of this hypothesis, we found no significant change in the expression of carbonic anhydrases 2, 9, or 14 transcripts by RNA sequencing (Supplementary Fig. 2e), implying the extracellular acidification is not primarily driven by a change in the equilibrium between CO_2_ and bicarbonate (HCO_3_^−^).

To disentangle the contributions of MCT1 and MPC1/2 to reduced pyruvate metabolism in DOX-treated cardiomyocytes, we used MPC1- and MPC2-specific siRNA to prepare HCM with selective inhibition of MPC1/2 (Supplementary Fig. 2a, f). These cells demonstrated a 25% reduction in [3-^14^C]pyruvate uptake compared to the scrambled control cells (*p* < 0.0001; Fig. 2d), although there were no changes in the expression of MCT1, MCT4, LDHA, LDHB, ALT, or the PDH complex (Supplementary Fig. 2g). A comparison between these results and the changes induced by DOX or UK5099 (Fig. 2c) suggests that deficits in DOX-induced pyruvate flux in cardiomyocytes are attributable to both impaired transport of pyruvate across the cell membrane (loss of MCT1 expression) and impaired transport across the mitochondrial membrane (loss of MPC1/2 expression).

One limitation of the uptake assay is that it is not possible to determine the chemical form of the ^14^C label after its addition to the well. There are three fates of the ^14^C label: it can be retained in the cell (as [3-^14^C]pyruvate, a TCA cycle intermediate, or another metabolite), released into the media (as [3-^14^C]lactate or [3-^14^C]pyruvate), or released as [^14^C]CO_2_, as shown in Fig. 2e. We hypothesized that one major difference between HCM consuming [3-^14^C]pyruvate in the TCA cycle and HCM deficient in MPC would be the production of [^14^C]CO_2_, which could be formed after multiple iterations of the TCA cycle (Supplementary Fig. 2h). Consequently, we performed a [^14^C]CO_2_ capture assay^34^ following incubation of HCM with [3-^14^C]pyruvate for 8 min. This experiment confirmed that lower activities of [3-^14^C]CO_2_ were evolved in the HCM with reduced MPC1/2 levels (*p* = 0.0002 for CTRL versus MPC1/2 siRNA; *p* = 0.0004 for CTRL versus UK5099; *p* = 0.0137 for CTRL versus DOX; Fig. 2f). Given the pH differences in the media between the control cells and those experiencing MPC inhibition, it is possible that our measurements underestimate the amount of [^14^C]CO_2_ produced by the control cells, as this product would be expected to be converted to bicarbonate to a greater extent than it would under more acidic pH. The total ^14^C counts, representing the sum of the [^14^C]CO_2_ captured, the cell-associated activity, and the remaining activity in the media, did not change (Supplementary Fig. 2i). Taken together, our findings indicate that the DOX-induced reduction in pyruvate metabolism in cardiomyocytes reflects impaired pyruvate transport and is partially mediated by MPC1/2.

### [3-^11^C]Pyruvate PET detects DOX-induced changes in cardiac pyruvate clearance at 4 weeks

**Fig. 3:**
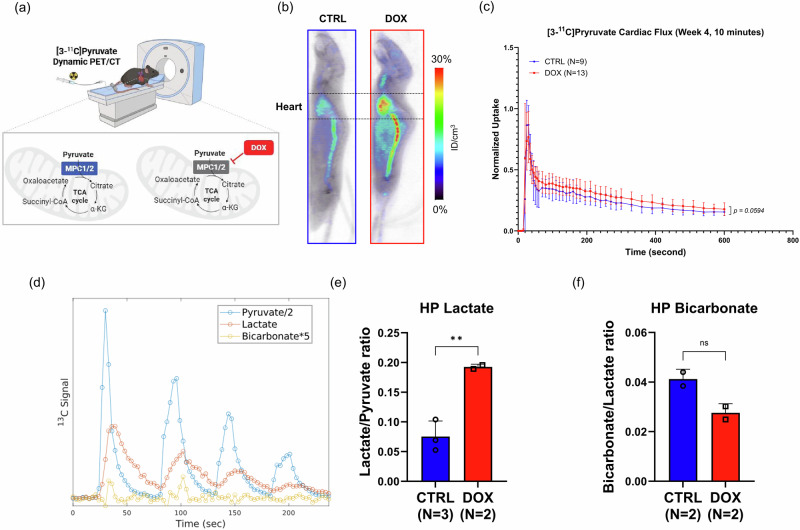
[3-^11^C]Pyruvate PET reveals DOX-induced alterations in cardiac pyruvate flux at 4 weeks. **a** Description of [3-^11^C]pyruvate dynamic PET/CT imaging. The lateral tail vein was cannulated for intravenous (i.v.) administration before imaging. **b** Representative summed sagittal PET images from the first 10 min post-injection in mice at the 4-week time point. **c** TACs over the initial 10-min PET acquisition period. Individual activity measurements were normalized to maximum uptake. **d** Representative multi-shot dynamic curves of HP [1-^13^C]spectroscopy in a control mouse heart. Intensities are scaled by the indicated factor to facilitate comparison. **e** Cardiac lactate/pyruvate ratio derived from HP [1-^13^C]pyruvate MR spectra. Mice (*n* = 3 in the CTRL group and *n* = 2 in the DOX group) were injected multiple times to provide a total of 10–12 independent measurements. **f** Cardiac bicarbonate/lactate ratios from the control (*n* = 2) and DOX (*n* = 2) groups. Data are presented as the mean ± s.d. *p < 0.05; **p < *0.01*. Statistical analysis was performed using the Mann–Whitney test between CTRL and DOX (**c**) or an unpaired t-test (**d**, **e**). PET positron emission tomography, TAC Time-activity curve, HP Hyperpolarized, MR magnetic resonance.

Given the potential significance of cardiac MPC as a target of DOX, we aimed to develop a method for assessing MPC levels in vivo. We hypothesized that measurements of the cardiac clearance of [3-^11^C]pyruvate by positron emission tomography (PET) (Fig. 3a) would highlight deficiencies in pyruvate transport and thereby permit MPC expression to be inferred. The rationale for this hypothesis was the difference in [^11^C]CO_2_ production between cells that utilize pyruvate in the TCA cycle and cells that do not. Based on our in vitro studies, as well as prior imaging studies with [1-^11^C]pyruvate in healthy hearts^19^, we anticipated rapid clearance of [^11^C]CO_2_ from cardiac tissue and a correspondingly rapid signal clearance in hearts without impaired transport. To test our hypothesis, we acquired 30 min dynamic PET images upon intravenous administration of [3-^11^C]pyruvate to mice exposed to DOX (*n* = 13) and age-matched controls (*n* = 10) at the 4-week time point (Supplementary Fig. 3a). Summed sagittal-axis PET images over the first 10 min post-injection showed a markedly enhanced [3-^11^C]pyruvate signal in the DOX group at 4 weeks compared to the control group (Fig. 3b). Consistent with PET images and [3-^13^C]pyruvate metabolomics, the time-activity curves (TACs) drawn using regions-of-interest (ROIs) over the whole heart from the 10-min acquisitions—normalized to peak uptake—highlighted a slower clearance of activity from the hearts of mice in the DOX group (Fig. 3c). Normalization to peak uptake accounts for differences in injected activity and comparisons of clearance rates minimize the effect of heart size on total tracer uptake. To quantify the difference in clearance rate, we focused on the interval from 2 to 10 min post-injection. Hyperpolarized MRI studies indicate that pyruvate washes out from the ventricle in mice after approximately 60–90 s^35,36^. As such, our window should primarily reflect signal clearance from cardiac muscle due to metabolism of [3-^11^C]pyruvate by cardiomyocytes. A monoexponential decay function was fit to the curve, and we compared the decay constant, *k*, between the mice in the DOX group (*n* = 13) and the control animals (*n* = 9). We established a minimum threshold of *R*^2^ > 0.8 as our acceptance criterion and rejected one animal from the control group for which the curve fitting did not reach this threshold. The decay constant for the mice exposed to DOX, 0.00266 ± 0.00102, was significantly lower (*p* = 0.0117) than the decay constant for the control mice, 0.00409 ± 0.00123. This translated to an increased transit time over this interval of 323 ± 29 s in the animals exposed to DOX (*n* = 13) compared to 312 ± 38 s in the control group (*n* = 9; *p* = 0.1803). The decay constant is proportional, and the transit time is inversely proportional, to the clearance rate of activity from cardiac tissue and supports the conclusion that [^11^C]CO_2_ is not generated as quickly in the hearts exposed to DOX.

As a further test of our hypothesis, we performed magnetic resonance spectroscopy (MRS) of hyperpolarized (HP) [1-^13^C]pyruvate MRI in the two groups at 4 weeks. In contrast to PET, MRS of HP pyruvate allows the metabolism of pyruvate to be directly observed in the mouse heart^35^. Although the HP tracer bore the isotopic label at the C-1 position rather than the C-3 position, the metabolism of these two isotopologues would be expected to be identical through PDH-catalyzed entry into the TCA cycle. We performed a multi-shot experiment to improve resolution (Fig. 3d) and observed a significant increase in the cardiac lactate/pyruvate ratio in the mice exposed to DOX (*p* = 0.0095; Fig. 3e). This is consistent with enhanced lactate production resulting from decreased utilization of pyruvate by the TCA cycle. Indeed, in a separate experiment using ^1^H NMR, we measured a higher lactate pool in cardiac tissues from mice in the DOX group compared to the control group (Supplementary Fig. 3b). In addition, the bicarbonate/lactate ratio was reduced by approximately 33% for the mice from the DOX group, although this difference did not reach statistical significance with our group size (*p* = 0.0713; Fig. 3f). The alanine/lactate ratio did not change between groups (Supplementary Fig. 3c). Collectively, these studies support the interpretation that slower cardiac [3-^11^C]pyruvate clearance corresponds to lower pyruvate oxidation and higher conversion to lactate.

### Recovery of MPC1/2 expression is coincident with accelerated cardiac growth

**Fig. 4:**
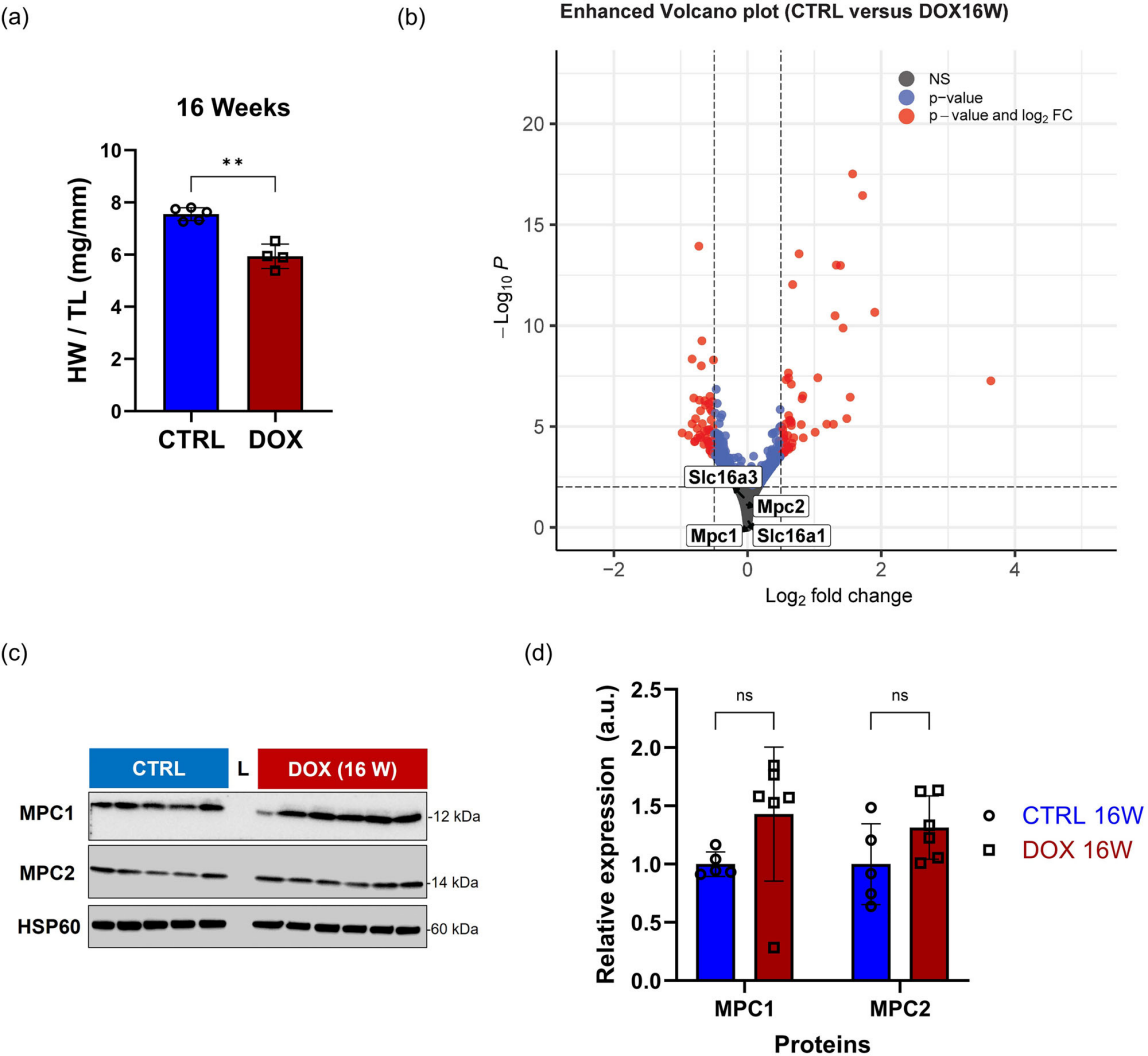
MPC1/2 expression rebounds 16 weeks after exposure in concert with the recovery of cardiac mass. **a** HW/TL ratio at 16 weeks after DOX exposure. **b** EnhancedVolcano plot from the bulk RNA sequencing at 16 weeks (Log2 fold change (FC) > | 0.5 | , FDR < 0.05). The Mpc1 and Mpc2 genes are highlighted. **c** Western blot analysis of MPC1 and MPC2 expression in cardiac tissue collected at 16 weeks. HSP60 was used as a reference. **d** ROI quantification of each protein expression level was performed using ImageJ. Data are presented as the mean ± s.d. **p < *0.01*. Statistical analysis was performed using an unpaired t-test (**a**) or two-way ANOVA (**d**). HW/TL heart weight-to-tibia length, FDR false discovery rate.

Exposure to DOX is known to induce cardiac atrophy followed by a period of accelerated growth^29,37^. In other pre-clinical models, impaired pyruvate oxidation—as induced by loss of MPC expression—promotes hypertrophy and impairs cardiomyocyte survival^26,38^. Therefore, we sought to investigate whether pyruvate transport differs during the growth phase in the DOX model. For this purpose, we collected heart tissue from mice 16 weeks after the first administration of DOX. The heart-weight-to-tibia-length (HW/TL) ratio was 5.94 for these mice, compared to 7.55 for the controls (*p* < 0.0001, Fig. 4a). At 4 weeks, the ratios were 5.13 and 7.69, respectively. As such, the HW/TL ratio in DOX-treated tissues at 16 weeks demonstrated a 1.2-fold increase relative to those at 4 weeks of DOX exposure (*p* = 0.0023; Supplementary Fig. 4a). We correspondingly observed increased expression of markers of cardiomyocyte growth and cardiomyopathy, such as p53^39^ and p21^40^, as shown in Supplementary Fig. 4b. Bulk RNA sequencing analysis conducted at 16 weeks (false discovery rate; FDR < 0.05) for KEGG pathways reinforced the growth profile of the heart, as shown in Supplementary Fig. 4c and Supplementary Table 6. Additionally, the EnhancedVolcano plot of the DEGs revealed neither Mpc1 (Log2FC = −0.00012) and Mpc2 (Log2FC = −0.02665) nor Mct1 (Log2FC = 0.00508) and Mct4 (Log2FC = −0.04813) to be significantly different between the mice exposed to DOX and the control animals (Fig. 4b). None of the 10 most down- and up-regulated DEGs were related to pyruvate metabolism (Supplementary Table 7). Similarly, and in contrast to the expression profile in the samples collected at 4 weeks, there were neither significant differences in MPC1 and MPC2 protein expression nor MCT1 and MCT4 expression between the mice in the DOX and control groups at 16 weeks (Fig. 4c, d and Supplementary Fig. 4d). Expression of other key components of pyruvate metabolism, such as LDHA, LDHB, and ALT, was also comparable between the groups, as shown in Supplementary Fig. 4d. A similar trend was evident in genes encoding key proteins involved in β-oxidation, whose expression significantly increased at 16 weeks compared to 4 weeks (Supplementary Fig. 4e), albeit without recovering to the expression levels in the control hearts.

### Cardiac [3-^11^C]pyruvate clearance suggests that pyruvate metabolism trends towards sustained functional impairment

**Fig. 5:**
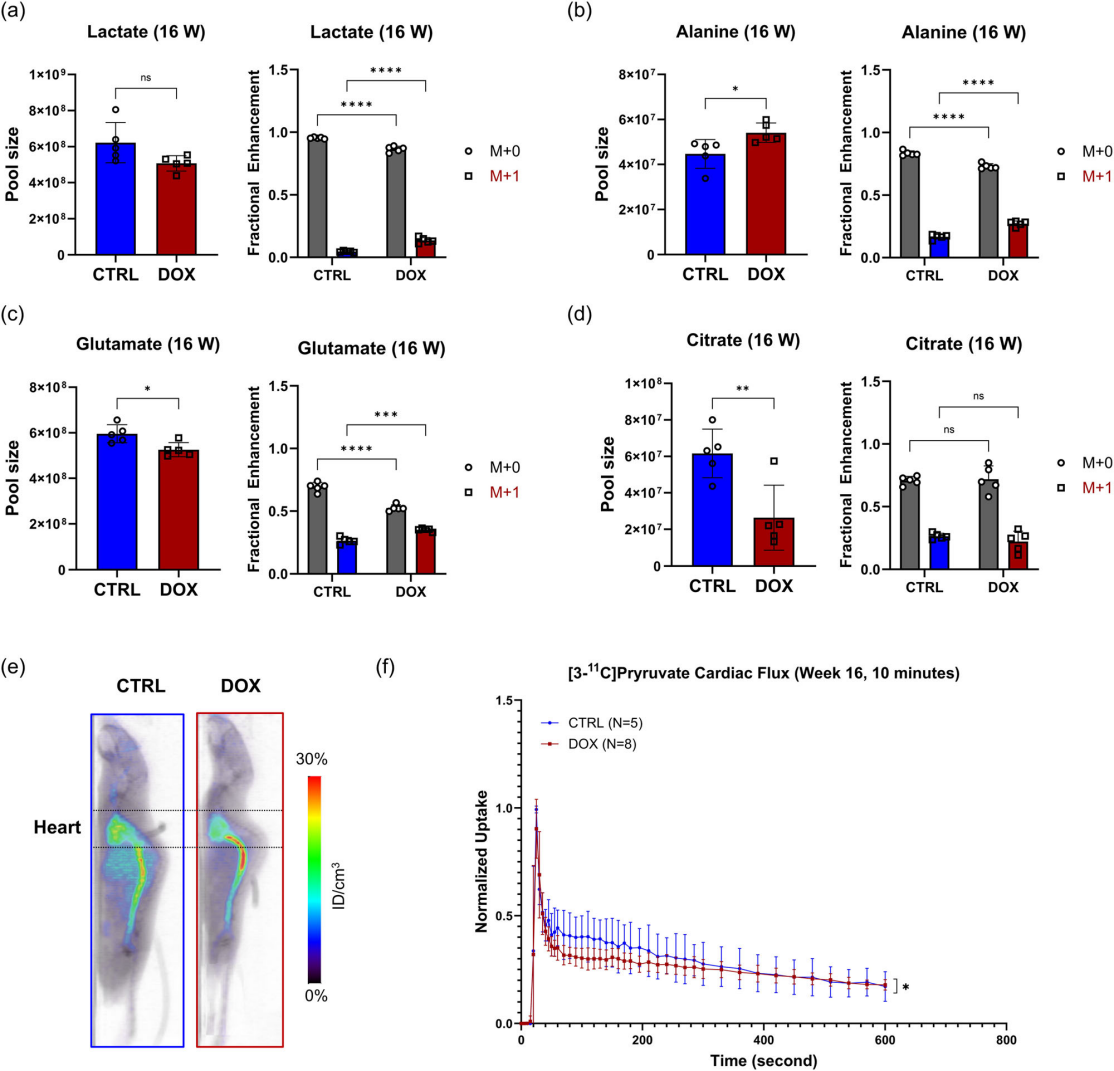
[3-^11^C]Pyruvate PET imaging captures changes in cardiac pyruvate metabolism. Comparison of total pool size and M + 0 and M + 1 fractional enrichments of **a** lactate, **b** alanine, **c** glutamate, and **d** citrate assessed during [3-^13^C]pyruvate stable isotope tracing metabolomics experiments. The experiment was performed 16 weeks after the first DOX exposure. Heart samples were collected 10 min post-injection of [3-^13^C]pyruvate. **e** Representative summed sagittal PET images from the first 10 min post-injection of [3-^11^C]pyruvate in mice at the 16-week time point. **f** TACs constructed using an ROI over the whole heart over the initial 10-min acquisition period. Activities at each measurement were normalized to maximum uptake. Data are presented as the mean ± s.d. *p < 0.05; **p < *0.01*; ***p < 0.001; ****p < 0.0001. Statistical analysis was performed using a two-way ANOVA (**a**–**d**) or the Mann–Whitney test (**c**). TCA Tricarboxylic acid, PET positron emission tomography, TAC time-activity curve, ROI Region-of-interest.

Having observed the recovery of MCT1 and MPC1/2 expression at 16 weeks, we sought to determine how these changes affected metabolism. We conducted stable isotope tracing metabolomics at 16 weeks using the same methodology as at 4 weeks. In contrast to the 4-week time point, we detected significantly increased M + 1 fractional enhancement of lactate (*p* < 0.0001), alanine (*p* < 0.0001), and glutamate (*p* = 0.0002) in the animals exposed to DOX, while fractional enhancement of citrate was comparable between the two groups (Fig. 5a–d). The total pool of lactate was comparable to the control samples, but the alanine pool was significantly greater (*p* = 0.0295), and the glutamate and citrate pools were significantly lower (*p* = 0.0157 and *p* = 0.0086, respectively) in the hearts exposed to DOX (Fig. 5a–d). Although we observed substantially increased fractional incorporation of the ^13^C label into metabolites associated with the TCA cycle, with M + 1 fractional enhancement of key metabolites, including aconitate, fumarate, malate, oxoglutarate, and succinate, comparable or significantly greater than the corresponding controls (Supplementary Fig. 5a), the total pool size of these metabolites—and the total of all metabolites detected—was significantly lower (*p* = 0.0035) in the mice exposed to DOX than the controls (Supplementary Fig. 5b). Consequently, the flux of [3-^13^C]pyruvate through the TCA cycle was lower in the hearts of the mice exposed to DOX compared to the hearts of the control group. Collectively, these findings indicate that pyruvate is primarily diverted away from the TCA cycle into the cytosolic metabolites lactate and alanine.

When these mice were imaged by [3-^11^C]pyruvate PET, we observed similar cardiac signal between the two groups in the summed image (Fig. 5e) but significant differences in the corresponding time-activity curves (*p* = 0.0473). Clearance from the whole heart from 0 to 2 min post injection, when ventricular washout is a major component of signal clearance, was notably faster in the mice exposed to DOX (Fig. 5f). However, in the interval between 2 and 10 min post injection, the constant of the exponential decay function fitted to the cardiac tissue measurements in the DOX group (*n* = 8; 0.00074 ± 0.00010) was markedly lower than the constant for the curve in the control group (*n* = 5; 0.00306 ± 0.00191), although this difference did not reach significance (*p* = 0.0534). All of the regressions were fit with *R*^2^ > 0.8. The corresponding transit times for the DOX and control groups in the 2–10 min interval were 380 ± 7 s and 321 ± 39 s, respectively (*p* = 0.0286). Despite these differences at early timepoints, as we observed in the 4-week cohorts, the cardiac time-activity curves for [3-^11^C]pyruvate trended towards convergence after 30 min (Supplementary Fig. 5c). Taken together, these results indicate that pyruvate metabolism trends toward functional impairment in the mice exposed to DOX at 16 weeks compared to their control cohort despite recovery of protein and gene expression levels.

In concert with these changes in pyruvate metabolism, we observed increased cardiac uptake of [^18^F]FDG in the mice exposed to doxorubicin at 16 weeks compared to the control group (Supplementary Fig. 5d). Mean uptake at 30 min p.i. for the DOX group (*n* = 6) was 9.1 ± 2.2%ID/cm^3^ while uptake at the corresponding timepoint in the control hearts (*n* = 6) was 6.1 ± 1.9%ID/cm^3^ (*p* = 0.0318). These findings reinforce a reprogrammed metabolism characterized by reduced pyruvate uptake and increased conversion of pyruvate to lactate. Overall, our findings support the hypothesis that exposure to DOX causes both rapid and sustained changes in cardiac pyruvate metabolism by modulating pyruvate transport in cardiomyocytes. In this model, initial deficits in MCT1 and MPC1/2 expression contribute to decreased utilization of pyruvate by the TCA cycle. Even after MPC1/2 expression (and MCT1 expression) is restored, changes in pyruvate metabolism, in concert with increased glycolysis, are observable. Significantly, this dynamic process can be imaged in vivo using PET-based measurements of [3-^11^C]pyruvate cardiac clearance.

## Discussion

Healthy cardiac tissue generates the bulk of its energy from oxidizable substrates via processes that take place in mitochondria, which comprise a large part of the cardiomyocytes^41^. However, during the onset and progression of doxorubicin-induced cardiotoxicity, cardiac tissue undergoes significant molecular and metabolic remodeling necessitated by the emergence of mitochondrial dysfunction. Impaired mitochondrial function may ultimately lead to cardiomyocyte apoptosis and cell death^42,43^. In this context, we identified pyruvate as a key metabolite in a rodent model of cardiotoxicity. Pyruvate, the end-product of glycolysis, plays a pivotal role in mitochondrial ATP production after its entry into the TCA cycle via PDH or its conversion to oxaloacetate via pyruvate carboxylase. One of the major regulators of this process is the MPC, which is exclusively responsible for mitochondrial pyruvate import^44^ and reported by some groups to be a rate-limiting step in pyruvate oxidation in the heart^45^. As MPC can regulate both pyruvate oxidation and anaplerosis, it may contribute more significantly to cardiac metabolic reprogramming than PDH^23^. To this end, reduced expression or genetic knockout of MPC1/2 was recently shown to promote hypertrophic cardiomyopathy in murine models of heart failure^23–25^ while MPC1/2 abundance promoted survival of porcine cardiac tissue following ischemia-reperfusion^46^. Similarly, reduced MPC1/2 expression has been observed in failing hypertrophic human hearts^24^ and patients who failed to respond to left ventricular assist device implantation^26^. MPC-mediated changes in pyruvate transport have also been linked to poorer survival in prostate cancer^33^, glioblastoma^47^, and many other cancers^48^.

Despite this emerging evidence of the importance of MPC in hypertrophic cardiomyopathy, cancer, and other diseases, we are not aware of any prior attempts to image this target in live subjects. To this end, we exposed mice to systemically administered doxorubicin as a model system. Doxorubicin exposure at these dose levels results in an initial phase of cardiac atrophy that precedes reduced left ventricular ejection fraction (LVEF)^7,49,50^. In this context, it is significant that we observed decreased MPC expression and activity when atrophy, as assessed by HW/TL ratio, was more pronounced, while expression and activity recovered during a phase of accelerated cardiac growth. Crucially, we did not observe cardiomyocyte loss in the atrophied hearts^29^ and found that DOX exposure was sufficient to suppress expression of pyruvate transporters in cardiomyocytes (Fig. 2b). Impaired pyruvate transport was evident in our multi-modal approach. Stable isotope tracing, hyperpolarized MRI and PET imaging experiments support decreased pyruvate transport through MPC in the atrophy phase, although the possibility that pyruvate uptake through MCT1 is rate-limiting cannot be discounted. By contrast, changes in pyruvate metabolism during the growth phase, while not statistically significant when assessed by PET, may be more consistent with impaired transport through MCT1 and upregulation of glycolysis. These findings indicate that [3-^11^C]pyruvate PET detects disease-related impairment of pyruvate metabolism, although our method does not currently distinguish between impairment driven by MPC and impairment arising from other processes.

Intriguingly, we observed recovery of MPC and MCT expression in the cardiac tissue samples collected 16 weeks after initial exposure to DOX. Indeed, although these differences were not significant, expression of these mediators of pyruvate transport trended higher in the DOX group. Despite this observation, we found pyruvate metabolism to be impaired in the hearts exposed to DOX. Given the rapid clearance of signal from the whole heart that we observed in these mice by PET during the first 2 min post-injection of [3-^11^C]pyruvate and the increased M + 1 fractional enhancement of TCA cycle metabolites, albeit at a lower pool size, after administration of [3-^13^C]pyruvate, we hypothesize that pyruvate uptake via MCT is most impaired. MCTs are bi-directional transporters that primarily import and export pyruvate and lactate to support the energetic requirements of cells and maintain intracellular pH levels. We observed enhanced cardiac [^18^F]FDG uptake in the DOX cohort (Supplementary Fig. 5d). Increased [^18^F]FDG uptake often reflects increased glycolysis^51^, a phenomenon associated with accelerated cardiac growth and emerging heart failure^52^. As lactate is a major product of glycolysis, we hypothesize that the cardiomyocyte MCTs in the cardiac tissue exposed to DOX are primarily engaged with lactate export at the expense of pyruvate import. This would result in reduced cardiac uptake of [3-^11^C]pyruvate in spite of equivalent expression levels of MCT. Further work is required to test this hypothesis through direct measurement of cardiac lactate efflux.

We elected to use [3-^11^C]pyruvate for our studies because it is readily synthesized from [^11^C]methyl iodide and a commercially available derivative of glycine^53,54^, and its longer retention in cardiac tissue compared to [1-^11^C]pyruvate would enable PET measurements over a longer time interval. Significantly, [3-^11^C]pyruvate can only produce [^11^C]CO_2_ by undergoing multiple rounds of the TCA cycle, as shown in Supplementary Fig. 2f. As such, clearance of signal from the myocardium is likely to be distinguishable from ventricular clearance, even in rodent models. This rationale was previously used to support the use of [3-^11^C]lactate to measure myocardial lactate kinetics in porcine hearts^54^. [3-^11^C]Lactate was found to track the fate of lactate in the heart, where it is either oxidized or backdiffused, and its kinetics were best described using a 2-tissue, 4-compartment model. This kinetic model likely fits the observed clearance of the ^11^C label from the mouse hearts following administration of [3-^11^C]pyruvate, but we were unable to perform the necessary metabolite corrections and corrections for right ventricle spill-in and spill-out due to the small blood volume of the mice. For this reason, we elected to quantify the differences in the clearance of radioactivity from an ROI encompassing the whole heart by comparing the decay constants of an exponential function fitted to the TACs. The TACs are best described by a biexponential decay function comprising a rapid clearance component (*k*_fast_) and a slower clearance component (*k*_slow_). As a substantial amount of the signal decrease in the first 2 min is due to the ventricular washout^35,36^, we focused our analysis on the interval from 2 min to 10 min post-injection, when clearance is described by *k*_slow_ and primarily represents the myocardial component. This simplification highlights phenomenological differences in cardiac pyruvate metabolism between groups but is not sufficient to decouple effects due to MCT, MPC, and other major mediators of metabolism. In our 4-week cohorts, slower clearance of signal from the heart was evident using both *k*_slow_ and transit time, *τ*. Differences in transit time, which represents the area under the TAC, did not quite reach statistical significance due to 2 mice in the control group whose τ was greater than 350 s (versus a mean value for the remaining 7 mice of 298 s). However, in contrast to the 4-week time point, the mice exposed to DOX exhibited a significant increase in *τ* compared to the control cohort, while the difference in *k*_slow_ did not reach significance (*p* = 0.0534) with our group sizes. While valuable as a metric for assessing the clearance of radioactivity from tissue, transit time—especially over a smaller time interval—is more sensitive than curve fitting to noise within the tissue activity measurements because the integral is normalized by the single highest activity value detected in the corresponding time interval. Therefore, comparison of differences in the decay constants is likely a more robust quantitative metric for assessing cardiac radioactivity clearance by PET.

At the 4-week time point, the difference in cardiac clearance rates following administration of [3-^11^C]pyruvate could be attributed to decreased [3-^11^C]lactate export or decreased [^11^C]CO_2_ production by the hearts exposed to DOX. Our MRI experiments highlight increased lactate production by the hearts of mice exposed to DOX (Fig. 3e and Supplementary Fig. 3b), although these differences were not statistically significant in our metabolomics experiment. This likely reflects differences in detection sensitivity between the analytical techniques and may also be a consequence of the time points at which the measurements were performed: within 1 min p.i. for the MRI experiment and 10 min p.i. for the metabolomics experiment. Furthermore, the MRI acquisition was performed on awake and restrained mice, potentially introducing a stressed state. However, given the short acquisition time (1 min) and the fact that these imaging experiments are relative, with empirically determined metabolite ratios compared between two groups of mice imaged under the same conditions at a time point at which we previously found cardiac function to be preserved^29^, it is likely that this imaging protocol minimally affects our interpretation of the findings. Despite increased cardiac lactate production in the DOX group, especially in the first minute post injection, our experiments do not support its prolonged accumulation in these hearts. MCT4 expression does not differ in cardiac tissue between the two groups (Supplementary Fig. 1e), and impaired lactate export by hearts exposed to DOX is inconsistent with our in vitro experiments (Fig. 2a, c, d). Moreover, we do not observe significantly slower cardiac ^11^C signal clearance in the hearts exposed to DOX at the 16-week time point, even though lactate production is enhanced compared to the 4-week time point (Fig. 5a).

We therefore interpreted the slower clearance of radioactivity in the DOX group at 4 weeks, evident in the PET imaging, to primarily reflect differences in the rate of formation of [^11^C]CO_2_. Cardiomyocytes efficiently and irreversibly eliminate CO_2_ through high CO_2_ permeability and carbonic anhydrase activity^55^. The [3-^14^C]pyruvate uptake studies in cultured human cardiomyocytes (Fig. 2) confirm that evolution of [^14^C]CO_2_ differs between cells exposed to DOX and control cells and is consistent with our hypothesis. To reinforce this interpretation, we measured the cardiac bicarbonate-to-lactate (Bic/Lac) ratios in mice exposed to DOX and control animals at the 4-week time point by HP [1-^13^C]pyruvate MRI. This substrate bears the isotopic label on a different carbon atom to our PET probe, but the rapid release of [^13^C]CO_2_ upon entry of [1-^13^C]pyruvate into the TCA cycle allowed use the formation of this metabolite as a means to distinguish between mitochondrial pyruvate oxidation and its metabolism by other pathways in a time course that is compatible with the hyperpolarization of [1-^13^C]pyruvate (T1 = 90 s in D_2_O^56,57^). Hyperpolarized [2-^13^C]pyruvate MRI has previously been used to measure labeling of downstream mitochondrial metabolites in rat hearts^12^ and could represent a valuable follow-up study for this work. Bicarbonate is formed when CO_2_ is hydrated by reaction with carbonic anhydrase. Bic/Lac ratios were typically lower in the mice exposed to DOX, as shown in Fig. 3f. Intriguingly, lower Bic/Lac ratios were associated with lower LVEF in human patients^16^, highlighting a possible relationship between pyruvate metabolism through MPC and cardiac function. The statistically significant increase in Lac/Pyr ratio in the hearts exposed to DOX (Fig. 3e) agrees with decreased flux of pyruvate through the TCA cycle and points toward a glycolytic phenotype in these hearts.

In cardiotoxicity, as in cardiomyopathies of other etiology, cardiomyocyte metabolic reprogramming precedes changes in cardiac structure and function^5,7,9,58,59^. Cardiac dysfunction, which may emerge months or years after the initial metabolic changes, is typically detected by echocardiography and defined in terms of parameters such as decreased LVEF. However, there remains a need to develop new methods of detecting incipient metabolic reprogramming by targeting specific biochemical pathways that are disrupted. In our previous work with the DOX model^29^, we demonstrated that significant cardiac dysfunction was not evident until 10 weeks after the first exposure to DOX. This timeline was comparable to other murine models in which the mice received a similar cumulative dose^7^. In this light, it is potentially significant that we observed reduced cardiac [3-^11^C]pyruvate clearance at 4 weeks. Future studies are required to determine whether early perturbation of myocardial pyruvate transport, as detected by [3-^11^C]pyruvate PET, predicts changes in LVEF or other functional parameters. These studies could confirm this technique to be an imaging biomarker of cardiotoxicity or other cardiomyopathies.

The major limitation of our approach is that non-invasive measurement of cardiac radioactivity clearance rates cannot necessarily isolate the effect of individual molecular targets. Our goal was to identify a method of non-invasively assessing MPC protein expression because decreased protein, but not gene expression, has been detected in cardiac tissue samples taken from patients with HFrEF^60^. We present evidence that the decreased pyruvate flux and cardiac clearance of [3-^11^C]pyruvate in the 4-week DOX group reflect decreased expression of MPC1 and MPC2, but we cannot definitively rule out the contribution of other enzymes and transporters to clearance patterns. Most significantly, expression of MCT1 mirrored that of MPC1/2 on a population level in our model, although individual differences in MCT1 expression might be responsible for the lower pool size and M + 1 fractional enrichment of lactate observed in a subset of the mice (*n* = 3) exposed to DOX (Fig. 1g). Conversion of HP [1-^13^C]pyruvate to [1-^13^C]lactate and other downstream metabolites is rate-limited by MCT1-mediated transport across the plasma membrane in cancer cells^61^, and the same kinetics likely apply to cardiomyocytes. One potential advantage of PET imaging in this context is that the tracer is administered in nanomolar concentrations, rather than the supra-physiological amounts required for HP [1-^13^C]pyruvate MR imaging and stable isotope tracing. As such, the likelihood of saturating transporters or disrupting metabolic equilibria is likely lower. Moreover, the longer half-life of carbon-11 compared to hyperpolarized carbon-13 enables the clearance of radioactivity from the heart to be studied for many minutes, thereby decoupling initial extraction from blood (i.e., transport through MCT) and metabolism within cardiac tissue (i.e., transport through MPC). As a result, the PET time-activity curves might more closely reflect MPC expression levels than MCT levels in some disease models. In addition, despite significantly lower levels of LDHB, the more prevalent isoform of LDH in the heart, we did not observe any impairment of pyruvate and lactate interconversion. This apparent contradiction can be explained by the observation that LDHB—and many of the enzymes involved in pyruvate metabolism—are subject to post-translational modification and substrate or product inhibition^62–64^.

A second limitation with respect to our desire to image MPC expression is the finding that [3-^11^C]pyruvate metabolism in the DOX-exposed hearts at 16 weeks differed compared to the controls, even though neither MCT1 nor MPC1/2 expression was significantly different in the hearts exposed to DOX. While we have put forward potential explanations for this discrepancy, this example nevertheless highlights the challenge of relating pyruvate flux measurements to changes in MPC protein expression levels alone. Indeed, in our experimental model, inference of MPC expression by [3-^11^C]pyruvate PET is possible only at early stages of metabolic reprogramming, i.e., before cardiomyocytes increase reliance on glycolysis. Moreover, expression of many components of pyruvate metabolism are dynamic in this experimental model, making it even more difficult to isolate the effect of changes to specific markers. Although neither of these limitations are necessarily a drawback with respect to its clinical application, MPC nevertheless represents a challenging target for imaging because probes are necessarily exposed to a number of transporters and enzymes before encountering the desired target and require uptake into cells before target binding. When the probe is a natural substrate for these enzymes and transporters, such as [3-^11^C]pyruvate, the potential for its involvement in competing or confounding pathways is relatively high. Additional insight into the complex relationship between MPC expression and pyruvate metabolism could therefore be gained through the development of a probe for PET imaging that selectively binds to MPC. These efforts are ongoing in our laboratory.

Collectively, our findings support dynamic [3-^11^C]pyruvate PET as a method for imaging cardiac pyruvate metabolism in vivo. Early changes induced by doxorubicin, including cardiac atrophy and loss of MPC1/2 and MCT1 expression, result in reduced oxidation of pyruvate in mitochondria and reduced clearance of radioactivity from the heart following administration of [3-^11^C]pyruvate. Despite recovered expression levels of pyruvate transporters 16 weeks after exposure to DOX, a trend towards impairment of pyruvate metabolism was observed by [3-^11^C]pyruvate PET through decreased cardiac uptake in the first 2 min post-injection. Although further work is required to decouple the effects of MCT and MPC on [3-^11^C]pyruvate uptake and clearance and establish feasibility in other models of cardiomyopathy, this study supports the potential of [3-^11^C]pyruvate PET for detecting impaired pyruvate transport, which could serve as an early indicator of cardiotoxicity in cancer patients undergoing doxorubicin treatment and a precursor to cardiac dysfunction in patients with cardiomyopathy.

## Methods

### General

Doxorubicin (DOX) hydrochloride was purchased from Tocris Bioscience, USA and used without further purification. It was dissolved at a concentration of 0.75 mg/mL in sterile saline for injection (Hospira, USA) with the aid of sonication. The solution was stored in the dark at –20 °C for up to 24 h before use.

### Stable isotope tracing

Four or 16 weeks after initial DOX exposure, DOX-treated mice (*n* = 3 and *n* = 5, respectively) and age-matched control mice (*n* = 4 and *n* = 5, respectively) received an intravenous bolus injection of 1 M sodium [3-^13^C]pyruvate (Millipore Sigma). The total injection volume was 100 ± 5 µL. At 10 min post-injection, blood was removed by cardiac puncture, and the mice were sacrificed by cervical dislocation. The heart was excised, dried, weighed, and flash frozen in liquid nitrogen. Metabolites were extracted using 80% methanol. The extracts were dried down and then re-dissolved in water. Targeted LC/MS analyses were performed on a Q Exactive Orbitrap mass spectrometer (Thermo Scientific) coupled to a Vanquish UPLC system (Thermo Scientific). The Q Exactive operated in polarity-switching mode. A Sequant ZIC-pHILIC column (2.1 mm i.d. × 150 mm, particle size of 5 µm, Millipore Sigma) was used for the separation of metabolites. A 2.1 × 20 mm guard column with the same packing material was used for the protection of the analytical column. The flow rate was set at 150 μL/min. The mobile phases consisted of 100% acetonitrile for mobile phase A and 0.1% NH_4_OH/20 mM CH_3_COONH_4_ in water for mobile phase B. The chromatographic gradient ran from 85% to 30% A in 20 min, followed by a wash with 30% A and re-equilibration at 85% A. The raw data were processed using El-MAVEN (v0.12.0). Metabolites and their ^13^C isotopologues were identified on the basis of exact mass within 5 ppm and standard retention times. The fractional abundance of isotopically labeled metabolites was determined by determining the ratio of the peak intensity of ^13^C-labeled species to the peak intensity of the corresponding natural ^12^C metabolite. The fractional labeling of each metabolite was compared by two-tailed, unpaired t-test, with *p* < 0.05 considered statistically significant.

### Radiosynthesis

#### General

A 5 M KOH solution was prepared by dissolving KOH, ≥99.95% trace metals basis (Millipore Sigma) in a suitable volume of 18 mΩ H_2_O. Stock solutions of 5 mg/mL D-amino acid oxidase from porcine kidney (D-AAO; Millipore Sigma) and 5 mg/mL catalase from bovine liver (Millipore Sigma) were prepared in 18 mΩ H_2_O. A 1 M solution of HCl in dioxane was prepared by diluting 0.4 mL of a 4 M HCl/dioxane solution (Millipore Sigma) with 1.2 mL anhydrous dioxane (VWR). Stock solutions of 0.5 M and 0.05 M Tris-HCl, pH 8.5, were prepared by diluting 1 M Tris-HCl, pH 8.5 (VWR) with 18 mΩ H_2_O.

### Production of [^11^C]CH_3_I

[^11^C]CO_2_ was produced by a [^14^N(p,α)^11^C] transformation on a TR19 cyclotron (Advanced Cyclotron Systems, Inc.). The [^11^C]CO_2_ was converted to [^11^C]CH_3_I using a TracerLab FX_C_ Pro (GE Healthcare). Conversion of [^11^C]CO_2_ to [^11^C]CH_3_I took approximately 14 min. [^11^C]CH_3_I was trapped on an ascarite column and distilled into the reaction vial in a stream of N_2_ gas by heating the column to 250 °C.

### Synthesis of [3-^11^C]pyruvate

[3-^11^C]pyruvate was synthesized following published methods^53,65^, with small modifications. Briefly, *N*-(diphenylmethylene)-glycine *tert*-butyl ester (3.0 ± 0.1 mg, Millipore Sigma) was dissolved in 350 µL N,N-dimethylformamide (ThermoFisher) in a glass vial. Next, 10 µL 5 M KOH was added, and [^11^C]CH_3_I (approximately 6 GBq) was transferred to the vial. After the [^11^C]CH_3_I was trapped, the reaction was heated at 85 °C for 5 min. The intermediate was diluted with 10 mL H_2_O and passed through a pre-conditioned Sep-Pak C18 plus short cartridge (Waters). The cartridge was washed with 5 mL H_2_O and eluted into a clean glass vial with 1.6 mL 1 M HCl in dioxane. Deprotection was effected by heating the reaction at 130 °C for 5 min. The contents of the vial were taken up in 20 mL H_2_O and passed through a Bond Elut Jr SCX 1000 mg cartridge (Agilent Technologies) pre-conditioned with 10 mL H_2_O. The cartridge was washed successively with 5 mL H_2_O and 2 mL 0.5 M Tris-HCl, and D/L-[3-^11^C]alanine was eluted in 2 mL 0.05 M Tris-HCl, pH 8.5. The mixture was transferred to a ThermoMixer® C (Eppendorf) and 75 µL of the D-AAO solution and 5 µL of the catalase solution were added. The reaction was shaken for 6 min at 40 °C before acidification to pH < 2 with the addition of 1 M HCl. The contents were passed through a pre-conditioned Bond Elut Jr SCX 1000 mg cartridge (Agilent Technologies). The filtrate was adjusted to pH 5–6 by the addition of 1 M NaOH. The radiochemical purity of the product was determined by analytical radioHPLC by injection onto a Chirex 3126 (D)-penicillamine, 4.6 × 150 mm column (Phenomenex). The isocratic mobile phase was 1 mM CuSO_4_ set to a flow rate of 1 mL/min. The retention time, *t*_R_, of the final product was compared to the *t*_R_ of sodium pyruvate (11.5 min).

### Small animal [3-^11^C]pyruvate microPET/CT imaging experiments

Prior to administration of [3-^11^C]pyruvate, the lateral tail vein of each mouse was cannulated for intravenous (i.v.) administration. A 27 G × ½ inch, 8 cm catheter (SURFLO® Winged Infusion Set, USA) was utilized for the cannulation procedure. Following confirmation of proper catheter insertion using a small saline flush, the tubing was prefilled with sterile saline and capped. Mice were imaged at 4 weeks after initial DOX exposure (*n* = 13 treated animals and *n* = 10 controls) or 16 weeks after initial DOX exposure (*n* = 8 treated animals and *n* = 5 controls).

Cannulated mice were anesthetized under isoflurane (3.5% for induction, 1.5% for maintenance) and placed in pairs on the imaging bed. Prior to radiotracer administration, a CT acquisition was performed for anatomic co-registration and scatter and attenuation correction. The mice were administered 3.7-11.1 MBq [3-^11^C]pyruvate in a total volume of 100–150 µL. Imaging was performed using small animal microPET/CT (Siemens Inveon™, USA), and the 30-min acquisition began immediately upon tracer injection. The data were collected in list mode, histogrammed into 54 dynamic frames (12 x 5 s, 12 x 10 s, 8 x 15 s, 10 x 30 s, 10 x 60 s, and 2 x 300 s) and reconstructed using the OSEM-MAP algorithm.

### microPET/CT image analysis

The global time-activity curves (TACs) were plotted by performing a biphasic nonlinear regression for the 30 min acquisition and a monophasic nonlinear regression for the 2–10 min interval using GraphPad Prism. Consistent with recent recommendations^66^, goodness of fit was determined by the *R*^2^ value. A value of *R*^2^ > 0.8 was considered the threshold for a good fit, and TACs with *R*^2^ < 0.8 were excluded from the analysis. This resulted in the exclusion of 1 mouse from the control 4 W group, whose decay constant was more than 1 order of magnitude different from the other 9 mice in the same control cohort. The mean decay constant, *k*, for the 2–10 min interval was compared between groups. Statistical comparisons were performed between each pair (e.g., DOX and control) at the 4-week and 16-week timepoints by an unpaired, two-tailed t-test with Welch’s correction. *P*-values < 0.05 were considered statistically significant.

Tissue transit time (*τ*) in the heart was determined by dividing the area under the curve (AUC) of the TAC for the 2–10 min interval by the highest activity detected in this interval. The mean *τ* values and standard deviations for each of the four groups were determined. Statistical comparisons of transit times between DOX and control groups at each time point were performed by an unpaired, two-tailed t-test with Welch’s correction. *P*-values < 0.05 were considered statistically significant.

### Small animal [^18^F]FDG microPET/CT imaging experiments

[^18^F]FDG was purchased from PETNET (Siemens, USA) as a solution in saline. Sixteen weeks after initial DOX exposure, mice (*n* = 5 per group) were cannulated as described above, and 3.8–7.3 MBq [^18^F]FDG in 100 µL saline was administered via the cannula. The image acquisition and reconstruction were performed as above. The percent injected dose per cubic centimeter (%ID/cm^3^) at 30 min post injection was determined by quantifying the activity in a region-of-interest drawn over the whole heart using the CT image for anatomical reference using the AMIDE freeware package. Statistical comparisons between the two groups were performed by an unpaired, two-tailed t-test with Welch’s correction. *P* < 0.05 were considered statistically significant.

### Hyperpolarization of [1-^13^C]pyruvate

Thirty-five microliter neat [1-^13^C]pyruvic acid doped with 15 mM AH111501 trityl radical was polarized in a SpinLab hyperpolarizer (GE Healthcare, USA) at 5 T and 0.8 K with 139.88 GHz microwave irradiation. After at least 45 min of solid-state polarization buildup, the frozen sample was dissolved with 10 mL superheated (~400 K) D_2_O buffer containing 100 mM Tris and 1 mM EDTA for a final [1-^13^C]pyruvate concentration of 100 mM. The HP pyruvate solution was pH-neutralized by a stoichiometric quantity of 10 N NaOH in the receiver flask. Dissolution polarization levels were estimated by measuring T1 decay on an aliquot of HP dissolution with a 1 T Spinsolve spectrometer (Magritek). The molar concentration of the pyruvate solution was measured by ^13^C NMR at 11.7 T (Bruker; MSKCC NMR Core) with reference to a 100 mM [^13^C]urea standard.

### ^13^C Magnetic resonance spectroscopy

Animal experiments were performed in a 3 T Biospec MRI scanner (Bruker, USA) with a ^13^C/^1^H volume quadrature coil (RAPID MR). A T1-weighted FLASH sequence was used for ^1^H anatomic imaging of the heart. Pre-scans were performed while mice were under ~1.5% isoflurane anesthesia. Immediately before initiating the hyperpolarized [1-^13^C]pyruvate dissolution process, the isoflurane flow was turned off, and the mouse was allowed to wake up in the scanner. Mice (*n* = 3 for the control group, *n* = 2 for the DOX group) were restrained with three pieces of double-backed tape to prevent movement during the experiment and were not left awake under restraint for more than 5 min. The total time under isoflurane anesthesia was less than 30 min. For the ^13^C spectroscopy experiment, the scanner was set to continuously acquire slice-selective spectra with a 60° flip angle, 2048 spectral points, and 1280 Hz (39.9 ppm) bandwidth. The HP pyruvate bolus was split into multiple injections to enhance the number of replicates measured per HP pyruvate dissolution^67^. One hundred microliter of 100 mM HP pyruvate was injected via tail vein catheter four times at 35–40 s intervals to allow complete relaxation of the preceding HP pyruvate injection.

### MR image analysis

Dynamic spectra were zero-filled, phased, apodized with 5 Hz exponential decay, and baseline-corrected in MNova software (Mestrelab Research). Pyruvate (171 ppm), lactate (183 ppm), alanine (176 ppm), and bicarbonate (161 ppm) signals were then quantified by integration. Treating each injection of HP pyruvate substrate as a distinct measurement, dynamic area under curve (AUC) values were calculated by summing the metabolite integral intensities over the time-course of the pyruvate bolus. Metabolite AUCs were normalized by calculating lactate-to-pyruvate ratios and bicarbonate- or alanine-to-lactate ratios. Multiple measurements acquired in the same subject were averaged together, and the resulting mean values were statistically compared between groups by two-tailed, unpaired t-test, with *p* < 0.05 considered statistically significant. A threshold of 5 was set for the signal-to-noise ratio (SNR), which resulted in the exclusion of one control mouse from the lactate/bicarbonate ratio plot.

### ^1^H NMR measurement of tissue lactate pool size

Four weeks after initial exposure to DOX, mouse hearts (*n* = 4 per group) were dissected and snap frozen in liquid nitrogen. At least 100 mg tissue was transferred to pre-filled bead mill tubes (Fisher Scientific, USA) containing 400 µL 4% (w/v) perchloric acid and finely ground by a Fisherbrand™ Bead Mill 24 Homogenizer (Fisher Scientific, USA). Homogenates were centrifuged for 15 min at 14000 rpm and 4 °C, and the supernatant was transferred to a new tube containing 1 mL chloroform/tri-n-octylamine (78/22 v/v), followed by centrifugation for 15 min at 4000 rpm and 4 °C. The aqueous phase was transferred to a new tube and lyophilized overnight. Dried samples were reconstituted in D_2_O solvent containing 1 mM sodium trimethylsilylpropanesulfonate standard and transferred to 5 mm NMR tubes. ^1^H NMR spectra of metabolite extract samples were acquired in a 14.1 T spectrometer (Bruker; MSKCC NMR core). Spectra were processed, and metabolite peaks were quantified with Chenomx NMR suite software (Chenomx, Canada).

### Preparation of cardiac tissue for analysis

The mice were anesthetized by i.p. ketamine injection and perfused with phosphate-buffered saline (PBS) via the left ventricle at a constant pressure of 80 mmHg. To perform the molecular analysis, the whole hearts were homogenized by using liquid nitrogen and a mortar and pestle. The homogenized tissues were separated for RNA and protein extraction. The extracts were flash frozen in liquid nitrogen and stored at –78 °C until further use.

### RNA isolation

Frozen heart tissue fractions were collected and soaked in Trizol (Invitrogen, USA), and the RNeasy Fibrous tissue mini kit (Qiagen, USA) was used to isolate total RNA from heart tissues. Genomic DNA was removed by DNase I (Qiagen), and RNA was reverse transcribed using an iScript kit (Bio-Rad, USA). RNA extracts were validated prior to sequencing.

### Bulk RNA-seq library construction and data analysis

RNA libraries were sequenced with paired-end 50 bps on the NovaSeq 6000 Sequencer (Illumina, USA). The raw sequencing reads in BCL format were processed through bcl2fastq 2.20 (Illumina) for FASTQ conversion and demultiplexing. After trimming the adapters with cutadapt (version 1.18; https://cutadapt.readthedocs.io/en/v1.18/), RNA reads were aligned and mapped to the GRCm39 mouse reference genome by STAR (version 2.5.2; https://github.com/alexdobin/STAR)^68^, and transcriptome reconstruction was performed by Cufflinks (Version 2.1.1) (http://cole-trapnell-lab.github.io/cufflinks/)^69,70^. The abundance of transcripts was measured using Cufflinks, with fragments per kilobase of transcript per million mapped reads (FPKM) as the output. Raw read counts per gene were extracted using HTSeq-count version 0.11.2^71^. Gene expression profiles were constructed for differential expression, cluster, and principal component analyses with the DESeq2 package (https://bioconductor.org/packages/release/bioc/html/DESeq2.html)^72^. For differential expression analysis, pairwise comparisons were performed between two or more groups using parametric tests where read counts follow a negative binomial distribution with a gene-specific dispersion parameter. Corrected *p*-values were calculated based on the Benjamini-Hochberg method to adjust for multiple testing. For the differentially expressed genes (DEGs) analysis in the 4-week groups, *p* < 0.0001 was used as the substantial signifier of statistical significance among a total of 49,139 variables, and Log2FC (FC, fold change) > |1| was used to distinguish upregulated (Up) and downregulated (Down) DEGs, respectively. For the 16-week groups, those differentially expressed genes (DEGs) among a total of 49,135 variables with a false discovery rate (FDR) of less than 0.05 were divided into up- and down-regulated groups for analysis. The EnhancedVolcano plot was generated using R Studio for the overall distribution of DEGs.

### DAVID classification of DEGs

The Database for Annotation, Visualization, and Integrated Discovery (DAVID; https://david.ncifcrf.gov/) was employed to classify differentially expressed genes (DEGs) based on their biological functions. For the 4-week group, 389 upregulated and 1229 downregulated genes were analyzed, whereas 86 upregulated and 107 downregulated genes were assessed in the 16-week group. These DEGs were subjected to Gene Ontology (GO) enrichment analysis, focusing on biological processes (BP), as well as Kyoto Encyclopedia of Genes and Genomes (KEGG) pathway analysis. GO terms with a *p* < 0.05 were considered statistically significant.

### STRING database analysis

A protein-protein interaction (PPI) network was constructed to identify the associations between the target and related differentially expressed genes (DEGs) using the Search Tool for the Retrieval of Interacting Genes/Proteins (STRING) database (http://string-db.org/)^73^. GO terms and PPI networks with a *p* < 0.05 were considered statistically significant.

### Western blot analysis

Frozen heart tissue fractions were soaked in tissue protein extraction reagent (#78510, ThermoFisher, USA), supplemented with a protease inhibitor cocktail (#87786, ThermoFisher, USA) for protein extraction. Western blots were prepared and processed as previously reported^29^. The information on all primary and secondary antibodies can be found in Supplementary Table 8. The chemical luminescent signals were measured by the ChemiDoc imaging system (Bio-Rad, USA). Protein expression was quantified by drawing a region of interest (ROI) over the corresponding band using ImageJ software. HSP60 and vinculin were used as loading controls. Expression levels of these proteins did not change relative to each other or between control tissues and tissues exposed to DOX at either time point (Supplementary Fig. 6).

### In vitro [3-^14^C]pyruvate uptake in human cardiomyocytes

#### Cell culture

Human cardiomyocytes (HCMs) isolated from adult left ventricles (PromoCell, Heidelberg, Germany) were cultured according to the manufacturer’s instructions. Briefly, once the cultures reached 80–90% confluency, the cells were washed with PBS and refreshed with either control medium (DOX-free) or medium containing DOX or UK5099. Cells in the former group were incubated in media containing 0.1 µM DOX. The cells were incubated for 48 h, followed by a medium change with fresh medium. After an additional 48 h incubation, the medium was replaced again with 0.1 µM DOX-containing medium to complete the “two-hit” treatment^29^. Following a final incubation period of 48 h, the medium was removed and replaced with DOX-free control medium. All DOX-treated assays were performed on day 7. The second group of HCM was treated with 100 µM UK5099 for 72 h^33^. The control cells received an equal volume of dimethyl sulfoxide (DMSO).

### Silencing of MPC1 and MPC2

MPC1 and MPC2 siRNA were used to downregulate MPC1/2-specific expression in HCMs. Pre-designed siRNA products targeting MPC1 (#4392420, ID s28488) and MPC2 (#4392420, ID s24657), along with negative control siRNA (#4390843, scramble) were purchased and 50 nM of MPC1 and MPC2 siRNA and 100 nM of scramble siRNA were mixed with Lipofectamine RNAiMAX (#13778100) or Lipofectamine Stem (STEM00008) and Opti-MEM (#31985070) according to established protocols^74^. The siRNA mixture was applied to HCMs for 24 h, followed by replacement with fresh culture medium for an additional 24 h of incubation. MPC1 siRNA, sense; UGCUAUUCUUUGACAUUCAtt/antisense; UGAAUGUCAAAGAAUAGCAac. MPC2 siRNA, sense; UCACUUGUAAUUAUUCCAAtt/antisense; UUGGAAUAAUUACAAGUGAgt. All materials for siRNA work were purchased from ThermoFisher, USA.

### [3-^14^C]Pyruvate uptake

All cells were seeded in 24-well plates. Each group of cells was washed with PBS and incubated with a Hanks’ Balanced Salt Solution (HBSS, 21-023-CV, Corning, USA)-based labeling medium containing sodium [3-^14^C]pyruvate (0.1 mCi/ml, ARC0220, American Radiolabeled Chemicals, USA) diluted 1:2000 and 0.5% fatty acid-free bovine serum albumin (BSA, A7030, Sigma-Aldrich, USA) in each well for 0, 2, 4, and 8 min at 37 °C. The cells were then washed twice with PBS and lysed with 1% sodium dodecyl sulfate (SDS). The radioactivity of the cell lysates was measured after dilution with scintillation buffer using a Liquid Scintillation Counter (Tri-carb 2910 TR, Perkin Elmer, USA). Cell uptake was corrected for activity added and normalized to the total protein concentration at the time of the assay (% of uptake/mg). Total protein concentrations were determined using the BCA protein assay kit (ThermoFisher, USA). Statistical analysis was performed using Pearson correlation coefficients from Fig. 2a (*R*^2^ = 0.9728), two-way ANOVA (Fig. 2b), and unpaired t-test (Fig. 2d).

### [^14^C]CO_2_ measurement

All cells were seeded in 35 mm culture plates. For the [^14^C]CO_2_ capture assay, a Whatman filter (Millipore Sigma, USA) was placed in the plate cap and moistened with 100 µL of 40% KOH, according to established procedures^34^. The cells were treated with sodium [3-^14^C]pyruvate as described above and incubated at 37 °C for 8 min. After incubation, the cells were washed twice with PBS and lysed using 1% SDS. The filter was suspended in scintillation buffer for counting on the Liquid Scintillation Counter. In parallel, cell lysates and the remaining supernatants were mixed with a scintillation buffer for radioactivity counting. Statistical analysis was performed using one-way ANOVA (Fig. 2f and Supplementary Fig. 2h).

### Cell viability

The viability of the HCM treated with DOX or UK5099 was determined using the trypan blue method. Briefly, after cell harvesting by trypsinization and centrifugation, cell pellets were resuspended in PBS. An equal volume of trypan blue solution (ThermoFisher, USA) was added to the suspension and gently mixed. The mixture was incubated for 3 min at room temperature. Subsequently, 10 µL of the stained cell suspension was loaded onto a hemocytometer and examined under a light microscope. Viable cells (excluding dye) and non-viable cells (blue-stained) were counted manually, and the percentage of viable cells was calculated. Statistical analysis was performed using one-way ANOVA.

### Cell counting

HCMs were seeded in 96-well plates. Seven days following DOX treatment, both control and DOX-treated groups were washed with PBS and incubated with a CCK-8 solution for 2 h. Subsequent steps were performed following the manufacturer’s instructions (#K1018, APExBIO, USA).

### Extracellular pH measurement

All cells were seeded in 24-well plates and treated with either DOX or UK5099 as described above. Control cells received an equal volume of DMSO. Each group of cells was washed with PBS and incubated in HBSS medium for 8 min at 37 °C. The supernatant was collected, and the pH was measured using pH-Test 4.5-10.0 indicator strips (VWR Chemicals, USA).

### Statistics

Statistical analyses were performed as described using GraphPad Prism.

### Ethics statement

All animal experiments carried out in this protocol were approved by the Institutional Animal Care and Use Committees (IACUC) of Weill Cornell Medicine and Memorial-Sloan Kettering Cancer Center (protocol 2019-0043).

## Supplementary information

Supplementary information

Supplementary Data 1

## Data Availability

Data are provided within the manuscript or supplementary information files. DICOM files for PET images are available from the authors upon request.
