# Supplementary material for: [3-11C]Pyruvate PET detects alterations in cardiac pyruvate metabolism induced by doxorubicin chemotherapy
Source: Npj Imaging. 2026 Apr 16;4:28. doi: 10.1038/s44303-026-00165-8 (PMC13087250; doi:10.1038/s44303-026-00165-8)

SUPPLEMENTAL INFORMATION

[3-^{11}C]Pyruvate PET detects alterations in cardiac pyruvate metabolism induced by doxorubicin chemotherapy

Chul-Hee Lee[1,2], Thomas Ruan[2,3], Shuvra Debnath[1,2], Anja S. Wacker[1,2], Grace Figlioli[3], John W. Babich,[1,2,4,5,#] Sadek A. Nehmeh[2], Kayvan R. Keshari[2,3], James M. Kelly[1,2,4,5*]

1 Molecular Imaging Innovations Institute (MI3), Weill Cornell Medicine, New York, NY 10021, USA

2 Department of Radiology, Weill Cornell Medicine, New York, NY 10021, USA

3 Department of Radiology and Molecular Pharmacology Program, Memorial Sloan Kettering Cancer Center, New York, NY 10065, USA

4 Citigroup Biomedical Imaging Center, Weill Cornell Medicine, New York, NY 10021, USA

5 Sandra and Edward Meyer Cancer Center, Weill Cornell Medicine, New York, NY 10021, USA

# Current address: Ratio Therapeutics, Boston, MA

Supplemental Figures

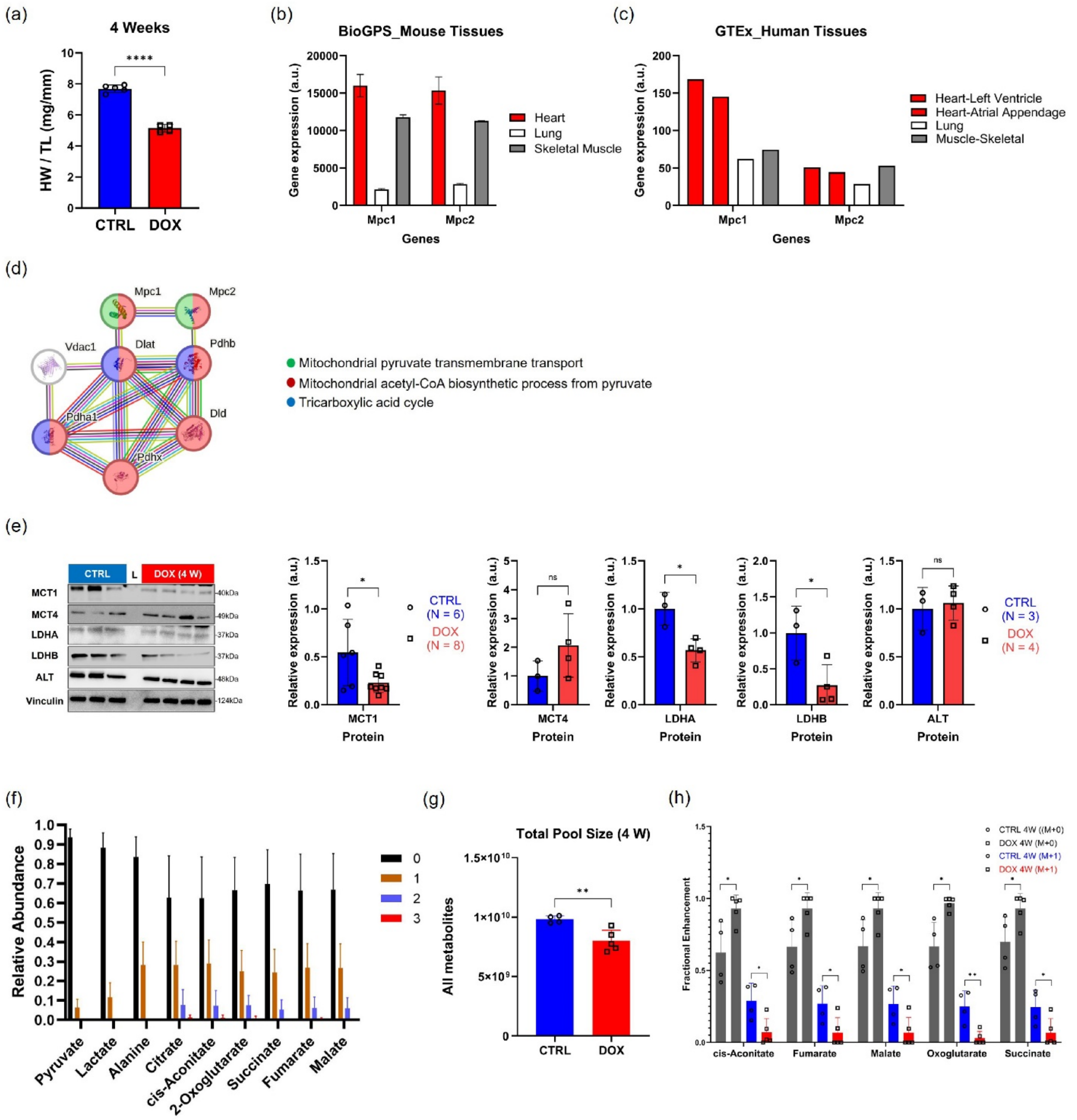

Supplemental Figure 1. ***DOX exposure reduces pyruvate metabolism in the mouse heart at 4 weeks.*** **(a)** HW/TL ratio at 4 weeks after DOX exposure. **(b)** Gene expression profiles of Mpc1 and Mpc2 in relevant mouse tissues were obtained from the BioGPS database. **(c)** Mpc1 and Mpc2 transcript levels in human tissues were obtained from the Genotype-Tissue Expression project (GTEx). Supplemental Table 3 displayed the numbers in detail. **(d)** STRING database from eight genes, including Mpc1/2 and their associated GO:BP = Acetyl-CoA biosynthetic process from pyruvate. **(e)** Western blot analysis of cardiac MCT1, MCT4, LDHA, LDHB, and ALT expression in the 4-week group. Vinculin was used as a reference. ROI quantification of each protein level was performed using ImageJ. **(f)** Incorporation of multiple isotopic labels into TCA cycle

intermediates by ^{13}C in healthy mouse hearts. **(g)** Comparison of the total metabolite pool size in the mice exposed to DOX with CTRL. **(h)** M+0 and M+1 fractional enrichments of other TCA cycle intermediates, including aconitate, fumarate, malate, oxoglutarate, and succinate. Data are presented as the mean ± s.d. * $p < 0.05$; ** $p < 0.01$. Statistical analysis was performed using an unpaired t-test (a), or two-way ANOVA (e,h). HW/TL = heart weight-to-tibia length; MPC = mitochondrial pyruvate carrier; DOX = doxorubicin; CTRL = control; GO:BP = gene ontology:biological process; STRING = Search Tool for the Retrieval of Interacting Genes/Proteins; MCT = monocarboxylate transporter; LDH = lactate dehydrogenase; ALT = alanine aminotransferase; TCA = tricarboxylic acid.

(a)

Human Cardiomyocytes	D0 – D2	D2 – D4	D5	D6	D7
CTRL	Media	Media	Media		Media
DOX	0.1 µM	Media	0.1 µM		Media
UK5099	Media	Media	100 µM UK5099		
MPC1/2 siRNA	Media	Media	Media	siRNA	Media

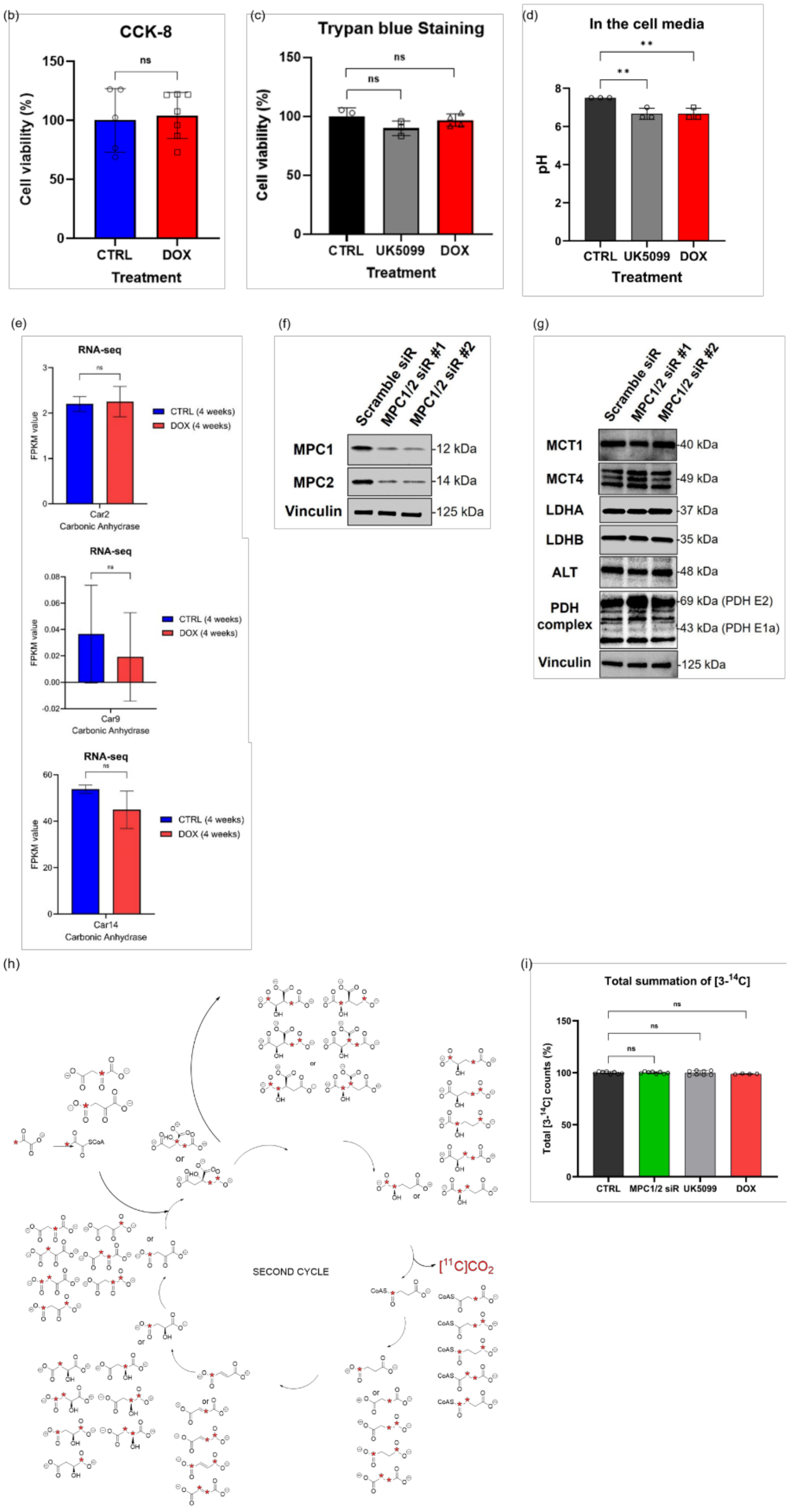

50

Supplemental Figure 2. ***DOX exposure impairs pyruvate uptake by downregulating MPC1/2 in HCM.*** **(a)** *In vitro* study design: HCMs were plated and assigned to control groups (vehicle or scramble siRNA) or experimental groups (DOX, UK5099, and MPC1/2 siRNA). Group conditions were optimized based on established protocol methods. **(b)** Cell viability according to DOX treatment was determined by the CCK-8 assay that detects the metabolic activity of cells. **(c)** Cell viability according to DOX or UK5099 treatment was determined by Trypan blue staining that detects the permeability of cell membranes from each of the cells. **(d)** The supernatant (cell media) from each group of cells treated according to the methods in Supplemental Fig. 2a was collected, and its pH was measured using pH-Test indicator strips to confirm lactate production. **(e)** Comparison of gene expression levels of carbonic anhydrase 2 (Car2; top), carbonic anhydrase 9 (Car9; middle), and carbonic anhydrase 14 (Car14; bottom) by RNA sequencing. **(f)** Western blot analysis of MPC1/2 expression in HCMs treated with siRNA. #1 and #2 mean the different lipofectamine conditions. Vinculin was used as a reference. **(g)** Western blot analysis of MCT1, MCT4, LDHA, LDHB, ALT, and PDH complex expression in HCMs treated with siRNA. Vinculin was used as a reference. **(h)** Incorporation of ^{13}C label into selected metabolites extracted from the hearts of healthy C57BL/6J mice administered [3-^{13}C]pyruvate. Hearts were excised 10 min p.i. Colored bars represent the number of ^{13}C labels per molecule and error bars represent standard deviations. **(i)** The total ^{14}C counts of each group of cells, representing the sum of the [^{14}C]CO2 captured, the cell-associated activity, and the remaining activity in the media. Data are presented as the mean ± s.d. * $p < 0.05$; ** $p < 0.01$. Statistical analysis was performed using unpaired t-tests (b,e) or one-way ANOVA (c, d, and i). HCM = human cardiomyocytes; PDH = pyruvate dehydrogenase.

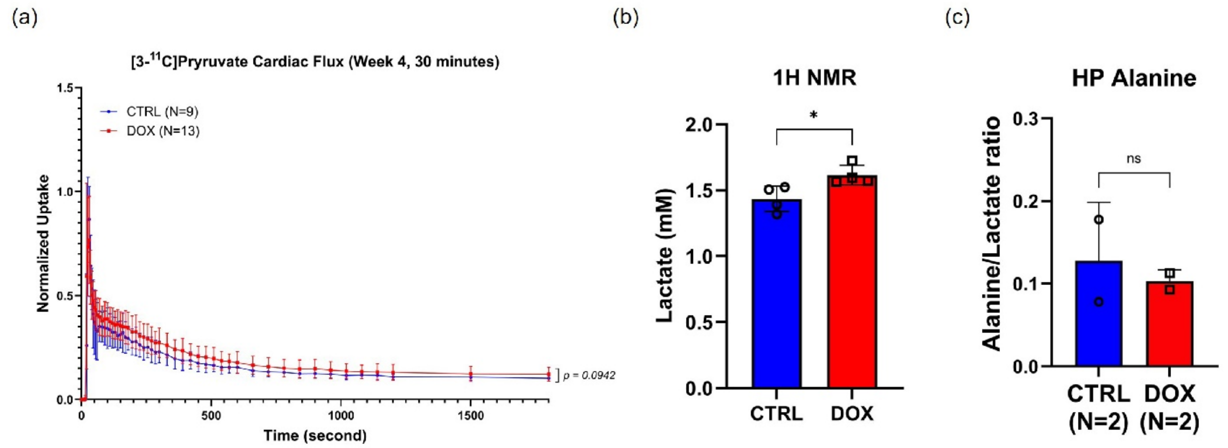

Supplemental Figure 3. ***[3-^{11}C]Pyruvate PET detects DOX-induced changes in cardiac pyruvate flux at 4 weeks.*** **(a)** Normalized time-activity curves for a VOI drawn over the whole heart of control mice (CTRL; n=9) and mice exposed to DOX (n=13). Images were acquired for 30 min following intravenous administration of [3-^{11}C]pyruvate. **(b)** Lactate pool in cardiac tissues from mice in the DOX group compared to the control group. ^{1}H NMR spectra of metabolite extract samples were acquired in a 14.1T spectrometer. Spectra were processed, and metabolite peaks were quantified with NMR software. **(c)** Cardiac alanine/lactate ratio derived from [1-^{13}C] pyruvate MRI. Mice (n=2 in the CTRL group and n=2 in the DOX group) were injected multiple times to represent 3–4 independent measurements per mouse. Data are presented as the mean ± s.d. * $p < 0.05$. Statistical analysis was performed using a Mann-Whitney test (a) or an unpaired t-test (b,c). VOI = Volume of interest; NMR = Nuclear Magnetic Resonance; HP = Hyperpolarized.

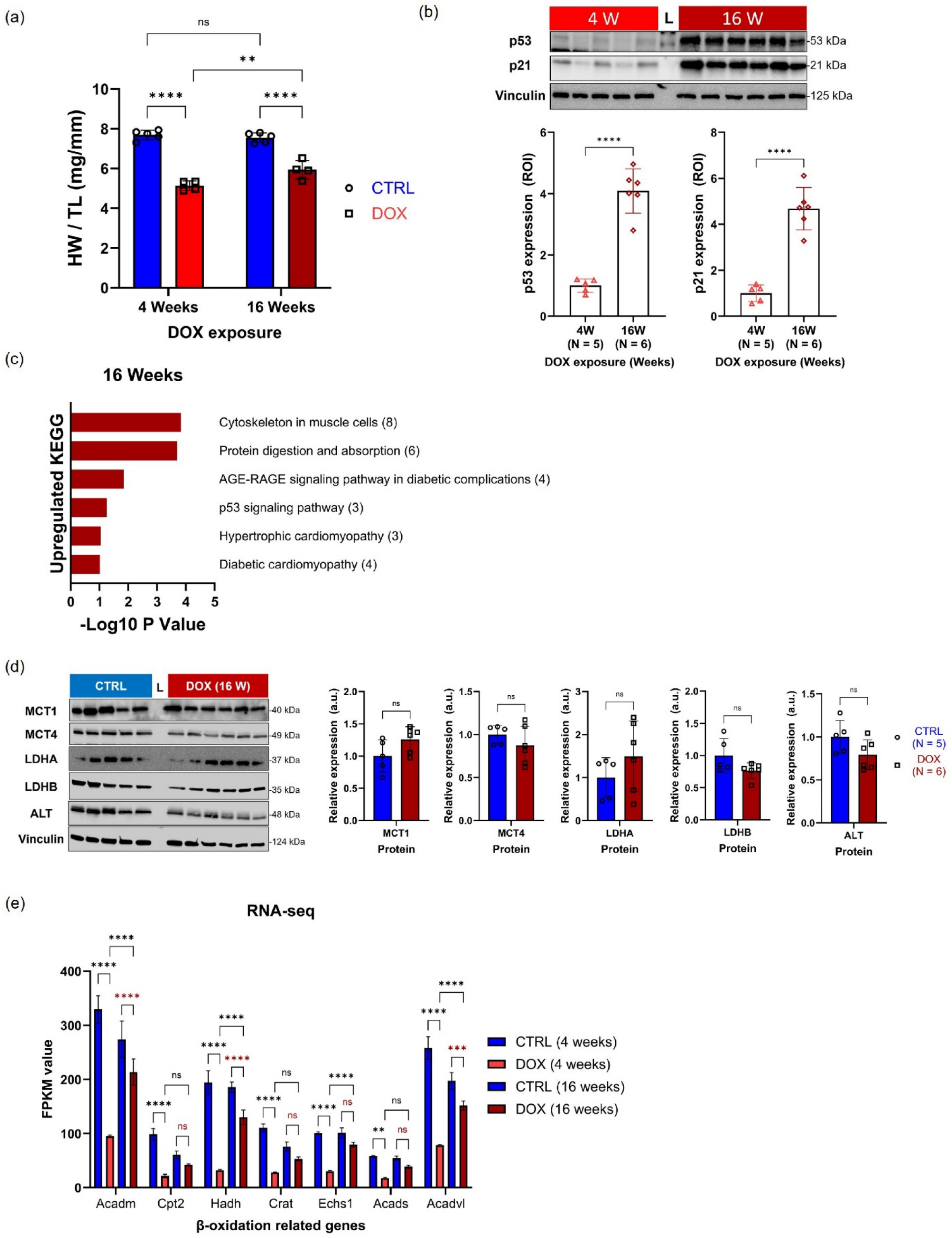

Supplemental Figure 4. ***Recovery of MPC1/2 expression is associated with accelerated cardiac growth.*** **(a)** HW/TL ratio in 4 and 16 weeks after DOX exposure. This graph was reorganized

using the Supplementary Fig. 1a and Fig. 4a. **(b)** Western blot analysis of cardiac p53 and p21 expression in the 16-week group. Vinculin was used as a reference. ROI quantification of each protein level was performed using ImageJ. **(c)** Significantly upregulated selected-KEGG pathways at 16 weeks after DOX exposure. Parentheses of (c) indicate the number of identified genes of each KEGG pathway. **(d)** Western blot analysis of cardiac MCT1, MCT4, LDHA, LDHB, and ALT expression in the 16-week group. Vinculin was used as a reference. ROI quantification of each protein level was performed using ImageJ. **(e)** Genes encoding key β-oxidation proteins were analyzed using RNA-seq-derived FPKM values from 4- and 16-week groups. Data are presented as the mean ± s.d. ** $p < 0.01$ and **** $p < 0.0001$. Statistical analysis was performed using two-way ANOVA (a, d, and e). HW/TL = heart weight-to-tibia length; KEGG = Kyoto Encyclopedia of Genes and Genomes; MCT = monocarboxylate transporter; LDH = lactate dehydrogenase; ALT = alanine aminotransferase. FPKM = Fragments Per Kilobase of exon per Million mapped fragments.

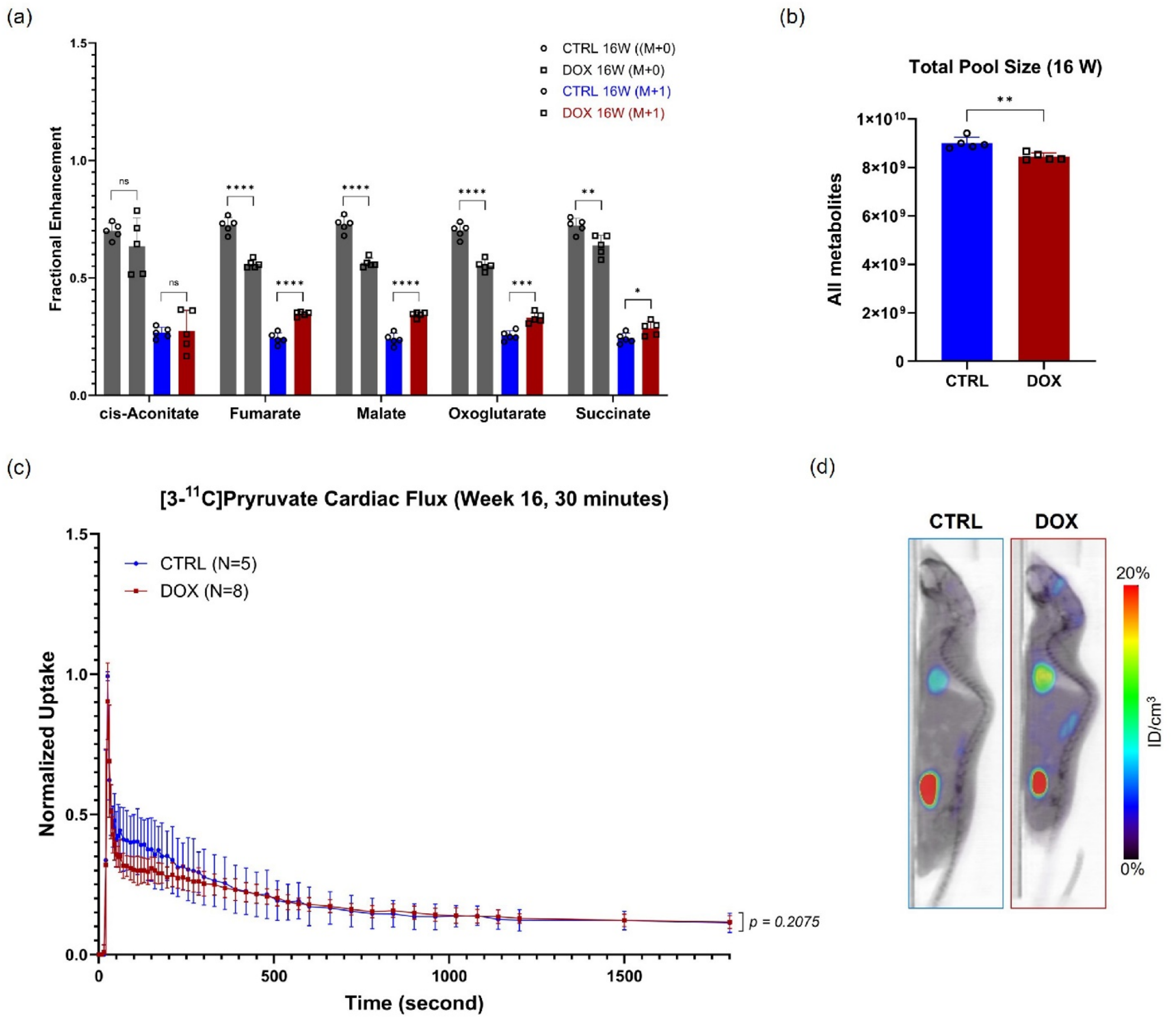

Supplemental Figure 5. ***Comparison between changes observed in [3-^{11}C]pyruvate PET imaging and shifts in pyruvate metabolism and β-oxidation components.*** **(a)** M+0 and M+1 fractional enrichments of other TCA cycle intermediates, including aconitate, fumarate, malate, oxoglutarate, and succinate at 16 weeks. (**b)** Comparison of the total metabolite pool size in the mice exposed to DOX with CTRL at 16 weeks. **(c)** Normalized time-activity curves for a VOI comprising the whole heart for mice exposed to DOX 16 weeks prior (n=8) or control mice (CTRL; n=5). Images were acquired for 30 min following intravenous administration of [3-^{11}C]pyruvate. **(d)** Representative sagittal [^{18}F]FDG PET images in mice at the 16-week time point. Images are presented as maximum intensity projections and represent the 5 min frame from 55-60 min post injection. Data are presented as the mean ± s.d. ** p < 0.01; *** p < 0.001; **** p < 0.0001. Statistical analysis was performed using two-way ANOVA (a), an unpaired t-test (b), or a Mann-Whitney test (c).

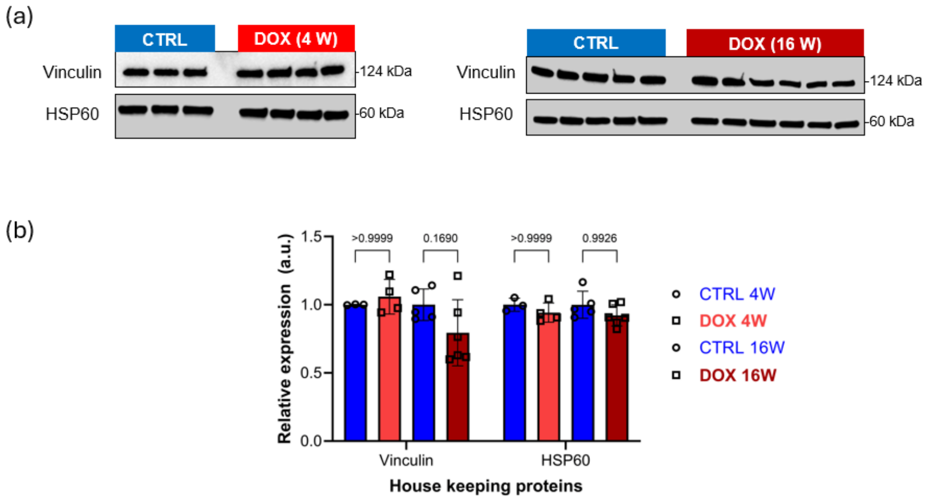

Supplemental Figure 6. ***Comparison of reference protein expression levels over time and according to treatment.*** **(a)** Western blots of vinculin and heat shock protein 60 (HSP60) expression in cardiac tissue collected from control mice (n=3) and mice exposed to doxorubicin (DOX) 4 weeks prior (left) or 16 weeks prior (right). **(b)** Quantification of vinculin and HSP60 expression. ROI quantification of each protein level was performed using ImageJ. Expression was compared between the two groups at each time point by unpaired t-test.

Supplemental Table 1. KEGG pathways by downregulated genes from the bulk RNA sequencing between CTRL and DOX of 4-week groups

**KEGG_PATHWAY (DOWN)**	**Term**	**Count**	**PValue**	**Genes**
mmu01100	Metabolic pathways	241	7.1E-44	PANK1, ENO3, GCSH, NAMPT, CHAC2, MLYCD, MMUT, ATP6V1E1, SEPHS2, MCCC2, PDHX, GSTK1, ACSL1, ENTPD5, OXSM, CMBL, ASRGL1, MTAP, ETHE1, HMBS, ACOT2, UQCRC1, ACOT1, SUCLG2, SUCLG1, LAP3, UQCRC2, ALG10B, LIPT2, HACD1, MGST3, PLA2G5, LDHB, LDHA, INPP5A, FDFT1, ST3GAL3, GSTM2, ACADSB, PRDX6, PAICS, CS, UCK2, ALDH6A1, ADI1, KYAT3, PDE3A, OGDH, ECHDC1, PPT2, GSTM7, GSTM5, PYGB, COX7B, HIBADH, GBE1, TECR, PIGP, ADK, HCCS, PYGM, GPT, PIGW, COX7C, GYS1, MCEE, ACADL, GUK1, IMPA2, SMPD1, RFK, ACADM, DLAT, ACADS, COX8A, COX8B, PCYT1A, TPI1, GSTO1, PGAM2, PLAAT3, HADHB, HADHA, ALDH5A1, BDH1, IVD, ADSSL1, L2HGDH, ALDOB, ALDOA, DLD, ECHS1, PRUNE1, DLST, HSD17B4, UQCR10, COX5B, HSD17B7, COX5A, SC5D, GPAT3, CBR4, NQO1, TIGAR, MDH1, CKM, MDH2, ATP5PB, PTGES2, GATC, COX6C, DHRS4, ALDH4A1, GPAM, NDUFAB1, ACAA2, NDUFA11, NDUFA12, NDUFA10, CPOX, HNMT, COX6A2, LCLAT1, ENPP4, KMT5A, HADH, DGAT2, SDHC, ACYP2, SDHD, SDHA, SDHB, COX6B1, PHOSPHO2, PGP, SMS, NDUFB10, UQCRB, NDUFB11, GLO1, AK1, GMPS, COX7A2, AK4, COX7A1, UQCRH, ACAT1, ESD, PGK1, COX11, SMYD1, CYC1, NDUFV3, NDUFV2, ATP6V1D, COX10, MPST, PDHA1, GOT1, EPHX2, IDH1, IDH2, GOT2, CRLS1, COQ5,

				QDPR, COQ3, AMACR, UQCRQ, CYCS, ACO2, NIT2, ACADVL, ALAS1, COX4I1, APIP, HK2, NADK2, GYG, DBT, ME1, IDH3B, UQCRFS1, ME3, HIBCH, PGM1, IDH3A, BCKDHA, PLA2G12A, GPX4, HMGCS1, BCKDHB, SORD, NDUFC2, NDUFC1, FH1, NDUFS8, PKM, ACOX1, NDUFS6, NDUFS5, EHHADH, CAT, NDUFS4, PCCB, NDUFS2, NDUFS1, GAPDH, DCTPP1, FBP2, NDUFB9, NDUFB8, NDUFB6, CARNMT1, NDUFB5, NDUFB4, NDUFB3, PDHB, HSD17B10, AGPAT3, CKMT2, UGP2, OXCT1, BPNT2, ACSS1, NDUFA9, NDUFA8, NDUFA7, DUT, FAHD1, IDH3G, NDUFA4, MMAB, NDUFA3, NDUFA1, SUOX, SUCLA2, NT5C1A, CHPT1, LPIN1, PFKM
mmu05415	Diabetic cardiomyopathy	76	1.3E-37	RYR2, COX7B, NDUFA11, NDUFA12, COX4I1, NDUFA10, ATP2A2, SLC2A4, COX6A2, COX7C, PPP1CB, GYS1, CPT2, MPC1, MPC2, UQCRFS1, CD36, CTSD, PDK2, COX8A, COX8B, NDUFC2, SDHC, NDUFC1, SDHD, SDHA, SDHB, AGTR1A, COX6B1, NDUFS8, NDUFS6, NDUFS5, NDUFS4, UQCRC1, PPIF, VDAC2, NDUFS2, VDAC1, NDUFS1, UQCRC2, SLC25A5, SLC25A4, GAPDH, NDUFB9, NDUFB8, NDUFB6, UQCRB, NDUFB10, NDUFB11, NDUFB5, NDUFB4, NDUFB3, COX7A2, PDHB, UQCR10, COX5B, COX5A, COX7A1, UQCRH, PLN, TNNI3, CYC1, NDUFV3, NDUFV2, NDUFA9, NDUFA8, NDUFA7, PDHA1, ATP5PB, NDUFA4, NDUFA3, NDUFA1, COX6C, AGT, UQCRQ, NDUFAB1
mmu00190	Oxidative phosphorylation	60	1.4E-35	COX7B, NDUFA11, NDUFA12, COX4I1, NDUFA10, COX6A2, COX7C, UQCRFS1, ATP6V1E1, COX8A, COX8B, NDUFC2, SDHC, NDUFC1, SDHD, SDHA, SDHB, COX6B1, NDUFS8, PPA2, PPA1, NDUFS6, NDUFS5, NDUFS4,

				UQCRC1, NDUFS2, NDUFS1, UQCRC2, NDUFB9, NDUFB8, NDUFB6, UQCRB, NDUFB10, NDUFB11, NDUFB5, NDUFB4, NDUFB3, COX7A2, UQCR10, COX5B, COX5A, COX7A1, UQCRH, COX11, CYC1, NDUFV3, NDUFV2, ATP6V1D, COX10, NDUFA9, NDUFA8, NDUFA7, ATP5PB, NDUFA4, NDUFA3, NDUFA1, COX6C, UQCRQ, NDUFAB1, CYCS
mmu01200	Carbon metabolism	53	5.3E-31	GPT, ENO3, HK2, GCSH, MCEE, ME1, IDH3B, ME3, MMUT, DLAT, ACADS, HIBCH, IDH3A, TPI1, PGAM2, SDHC, SDHD, SDHA, SDHB, FH1, PKM, ACOX1, CAT, PCCB, PGP, SUCLG2, SUCLG1, ALDOB, ALDOA, DLD, GAPDH, FBP2, ECHS1, DLST, PDHB, ACAT1, ESD, PGK1, ACSS1, PDHA1, MDH1, GOT1, MDH2, IDH3G, IDH1, IDH2, GOT2, CS, ALDH6A1, SUCLA2, OGDH, ACO2, PFKM
mmu05208	Chemical carcinogenesis - reactive oxygen species	68	9.7E-29	COX7B, NDUFA11, NDUFA12, COX4I1, NDUFA10, COX6A2, COX7C, AS3MT, UQCRFS1, COX8A, COX8B, GSTO1, NDUFC2, SDHC, NDUFC1, SDHD, SDHA, SDHB, COX6B1, NDUFS8, NDUFS6, NDUFS5, CAT, NDUFS4, UQCRC1, PPIF, VDAC2, NDUFS2, VDAC1, NDUFS1, UQCRC2, SLC25A5, SLC25A4, NDUFB9, NDUFB8, NDUFB6, UQCRB, NDUFB10, NDUFB11, NDUFB5, MGST3, NDUFB4, NDUFB3, COX7A2, UQCR10, COX5B, COX5A, COX7A1, UQCRH, CYC1, NDUFV3, NDUFV2, NDUFA9, NDUFA8, NQO1, GSTM2, NDUFA7, ATP5PB, NDUFA4, EPHX2, NDUFA3, NDUFA1, SOD2, COX6C, UQCRQ, NDUFAB1, GSTM7, GSTM5
mmu04260	Cardiac muscle contraction	37	4.2E-21	RYR2, COX7B, UQCRB, COX4I1, ATP2A2, COX7A2, ATP1A1, UQCR10, COX5B, COX7A1, COX7C, COX5A, COX6A2, UQCRH, SLC8A1, CACNG6,

				UQCRFS1, TNNI3, CACNA1S, CYC1, COX8A, COX8B, CACNA2D1, TNNC1, TPM1, COX6C, ATP1B1, TRDN, COX6B1, ACTC1, UQCRQ, MYL2, MYL3, UQCRC1, CASQ2, HRC, UQCRC2
mmu00020	Citrate cycle (TCA cycle)	23	1.0E-19	PDHA1, MDH1, MDH2, IDH3G, IDH1, IDH2, DLST, SDHC, PDHB, SDHD, SDHA, SDHB, FH1, CS, SUCLA2, OGDH, IDH3B, SUCLG2, SUCLG1, ACO2, DLAT, DLD, IDH3A
mmu00280	Valine, leucine and isoleucine degradation	25	1.2E-14	ECHS1, ACAA2, HIBADH, HSD17B10, ACAT1, MCEE, OXCT1, DBT, ACADM, MMUT, HADH, ACADS, HIBCH, BCKDHA, MCCC2, HMGCS1, BCKDHB, ACADSB, HADHB, HADHA, ALDH6A1, EHHADH, IVD, PCCB, DLD
mmu01212	Fatty acid metabolism	21	5.9E-10	ACADVL, HACD1, ECHS1, ACAA2, ACSL1, OXSM, TECR, HSD17B4, ACADSB, ACAT1, HADHB, HADHA, CPT2, ACADL, ACOX1, EHHADH, ACADM, PPT2, HADH, ACADS, CBR4
mmu00620	Pyruvate metabolism	16	3.7E-08	PDHA1, MDH1, MDH2, GLO1, ACYP2, PDHB, FH1, ACAT1, LDHB, LDHA, PKM, ME1, ME3, DLAT, ACSS1, DLD

Supplemental Table 2. GO:BPs analysis by downregulated genes from the bulk RNA sequencing between CTRL and DOX of 4-week groups.

GO:BP (DOWN)	Term	Count	PValue	Genes
GO:0032543	Mitochondrial translation	51	3.4E-39	MRPS16, MRPS14, FASTKD2, MRPS12, MRPL39, MRPL36, MRPL34, MRPL35, MRPL32, MRPL41, MRPL4, MRPL42, MRPL2, MRPL1, MRPL9, CHCHD1, MRPS27, MRPS24, MRPS22, MRPS23, MRPL49, MRPS2, MRPS21, MRPL45, MRPS7, MRPL46, MRPL43, MRPL44, MRPS18C, MRPL50, MRPL51, NOA1, MRPS35, MRPL18, MRPL19, MRPS31, MRPL16, MRPL14, MRPL12, MRPL57, MRPL13, MRPL10, MRPL54, MRPL55, MRPL11, NDUFA7, GATC, MRPL28, MRPL21, MRPL22, MRPL30
GO:0032981	Mitochondrial respiratory chain complex I assembly	35	6.3E-27	NDUFB9, NDUFB8, DMAC1, NDUFA11, NDUFB10, NDUFB6, NDUFB11, NDUFA12, NDUFB5, NDUFB4, NDUFA10, NDUFB3, NDUFB1, AIFM1, NDUFA9, NDUFA8, NDUFA3, NDUFA1, NDUFC2, NDUFC1, TIMM21, BCS1L, OXA1L, LYRM2, NDUFS8, NDUFS6, NDUFS5, NDUFAF4, NDUFS4, NDUFAB1, TMEM126A, NDUFS2, NDUFS1, NDUFAF1, TMEM126B
GO:0009060	Aerobic respiration	36	8.5E-27	NDUFB9, NDUFB8, NDUFA11, NDUFB10, NDUFB6, NDUFB11, NDUFA12, NDUFB5, NDUFB4, NDUFA10, NDUFB3, NDUFB1, MTFR1L, NDUFV3, NDUFV2, COX10, NDUFA9, NDUFA8, NDUFA7, MDH2, NDUFA3, NDUFA1, NDUFC2, NDUFC1, SDHB, OXA1L, NDUFS8, NDUFS6, NDUFS5, CAT, NDUFS4, NDUFAB1, NDUFS2, MTFR1, NDUFS1, FXN
GO:0042776	Mitochondrial ATP synthesis coupled proton transport	33	6.8E-25	NDUFB9, NDUFB8, NDUFA11, NDUFB10, NDUFB6, NDUFB11, NDUFA12, NDUFB5, NDUFB4, NDUFA10, NDUFB3, NDUFB1, NDUFV3, NDUFV2, NDUFA9, NDUFA8, NDUFA7, ATP5PB, NDUFA3, NDUFA1, NDUFC2, SDHC, NDUFC1, SDHD, SDHA, SDHB, NDUFS8, NDUFS6, NDUFS5, NDUFS4, NDUFAB1, NDUFS2, NDUFS1
GO:0006099	Tricarboxylic acid cycle	24	8.1E-22	PDHA1, MDH1, MDH2, MRPS36, IDH3G, IDH1, IDH2, DLST, SDHC, PDHB, SDHD, SDHA, SDHB, FH1, CS, SUCLA2, NDUFS4, OGDH, IDH3B, SUCLG2, SUCLG1, ACO2, DLAT, IDH3A

GO:0006631	Fatty acid metabolic process	48	9.9E-19	ACADVL, HACD1, PRKAA2, ECHS1, ACAA2, ECI1, TECR, HSD17B4, LPL, DBI, ABHD5, PLA2G5, ALKBH7, HSD17B10, ACAT1, CPT2, ACADL, UCP3, THEM4, CD36, ACADM, MLYCD, HADH, ACAD11, ACADS, CBR4, DECR1, LYPLA1, ACSL1, PTGES2, PNPLA8, OXSM, ECH1, ACADSB, ACSF2, HADHB, GNPAT, HADHA, AMACR, GPAM, ACOX1, NDUFS6, EHHADH, NDUFAB1, ACOT2, ACOT1, ECHDC3, CRAT
GO:0006120	Mitochondrial electron transport, NADH to ubiquinone	17	7.0E-15	NDUFB9, NDUFA8, NDUFB8, NDUFA7, NDUFB6, BDNF, NDUFA10, NDUFC2, COQ9, DNAJC15, NDUFS8, NDUFS6, NDUFS2, NDUFS1, NDUFAF1, NDUFV2, DLD
GO:0006629	Lipid metabolic process	89	5.3E-14	RAB7, ACADVL, LPGAT1, ACAA2, ECI1, TECR, HDLBP, VLDLR, LCLAT1, LACTB, AKR7A5, CPT2, ACADL, SMPD1, ACADM, MLYCD, HADH, ACAD11, ACADS, PLA2G12A, LYPLA1, DGAT2, PCYT1A, HMGCS1, GPX4, ACSL1, OXSM, BCKDHB, ECH1, PLAAT1, ACOT13, PLAAT3, PTGR2, HADHB, HADHA, BDH1, ACOX1, EHHADH, ACOT2, ACOT1, PLBD1, PLIN5, HACD1, PRKAA2, ECHS1, RETSAT, INSIG2, MGST3, HSD17B4, LPL, FITM1, FITM2, ABHD5, PLA2G5, HSD17B7, HSD17B10, AGPAT3, ACAT1, OXCT1, THEM4, SC5D, RDH14, GPAT3, SPTSSA, ACSS1, CBR4, COX10, DECR1, FDFT1, GSTM2, SLC16A1, PTGES2, PNPLA8, ADHFE1, EPHX2, CIDEA, CRLS1, ACADSB, PRDX6, ACSF2, NCEH1, GPAM, LPCAT3, NDUFAB1, ECHDC3, CHPT1, LPIN1, CRAT, NFE2L1
GO:0007005	Mitochondrion organization	24	1.2E-08	EPM2A, RAB3A, PTCD2, MTFP1, MTX2, CHCHD10, PHB, BCS1L, SOD2, PHB2, HSD17B10, NIPSNAP2, PRDX3, PINK1, CHCHD2, OPA1, NOA1, DNAJA3, MFN2, MTFR1, TMEM126B, FXN, COX10, SLC25A46
GO:0006086	Acetyl-CoA biosynthetic process from pyruvate	8	3.1E-08	PDHX, PDHA1, MPC1, MPC2, VDAC1, PDHB, DLAT, DLD

Supplemental Table 3. Summary of the 10 most downregulated and upregulated differentially expressed genes (DEGs) from cardiac tissue collected from mice 4 weeks after initial exposure to DOX or the control cohort.

**Down-regulated**	**Full name of genes**	**NCBI Gene Summary**
Lingo3	Leucine Rich Repeat And Ig Domain Containing 3	Predicted to be active in extracellular matrix and extracellular space
Gpr22	G Protein-Coupled Receptor 22	This gene is a member of the G-protein coupled receptor 1 family and encodes a multi-pass membrane protein
Strit1	Small Transmembrane Regulator of Ion Transport 1	Predicted to be involved in regulation of calcium ion transport and regulation of striated muscle contraction
Scn4b	Sodium Voltage-Gated Channel Beta Subunit 4	The protein encoded by this gene is one of several sodium channel beta subunits
Pln	Phospholamban	The protein encoded by this gene is found as a pentamer and is a major substrate for the cAMP-dependent protein kinase in cardiac muscle
Fabp3	Fatty Acid Binding Protein 3	The intracellular fatty acid-binding proteins (FABPs) belongs to a multigene family
Klhdc8a	Kelch Domain Containing 8A	This gene encodes a kelch domain-containing protein which is upregulated in cancer
Ak4	Adenylate Kinase 4	This gene encodes a member of the adenylate kinase family of enzymes
Efnb3	Ephrin B3	A member of the ephrin gene family, is important in brain development as well as in its maintenance
Hadh	Hydroxyacyl-CoA Dehydrogenase	The encoded protein functions in the mitochondrial matrix to catalyze the oxidation of straight-chain 3-hydroxyacyl-CoAs as part of the beta-oxidation pathway
**Up-regulated**	**Full name of genes**	**NCBI Gene Summary**

Lrrn4	Leucine Rich Repeat Neuronal 4	Predicted to be involved in long-term memory
Lrp2	LDL Receptor Related Protein 2	The protein encoded by this gene, low density lipoprotein-related protein 2 (LRP2) or megalin, is a multi-ligand endocytic receptor
Upk1b	Uroplakin 1B	The protein encoded by this gene is a member of the transmembrane 4 superfamily, also known as the tetraspanin family
Kcnh7	Potassium Voltage-Gated Channel Subfamily H Member 7	Voltage-gated potassium (Kv) channels represent the most complex class of voltage-gated ion channels from both functional and structural standpoints
Slc26a3	Solute Carrier Family 26 Member 3	The protein encoded by this gene is a transmembrane glycoprotein that transports chloride ions across the cell membrane in exchange for bicarbonate ions
Msln	Mesothelin	Mesothelin is a glycosylphosphatidylinositol-anchored cell-surface protein that may function as a cell adhesion protein
Hydin	HYDIN Axonemal Central Pair Apparatus Protein	This gene encodes a protein that may be involved in cilia motility
Sds	Serine Dehydratase	This gene encodes one of three enzymes that are involved in metabolizing serine and glycine
Cyp2s1	Cytochrome P450 Family 2 Subfamily S Member 1	The cytochrome P450 proteins are monooxygenases which catalyze many reactions involved in drug metabolism and synthesis of cholesterol, steroids and other lipids
Bmp3	Bone Morphogenetic Protein 3	This gene encodes a secreted ligand of the TGF-beta (transforming growth factor-beta) superfamily of proteins

Supplemental Table 4. Bulk tissue gene expression in humans for Mpc1 (ENSG00000060762.19) and Mpc2 (ENSG00000143158.11). The data source was determined by GTEx Analysis Release V10 (dbGaP Accession phs000424.v10.p2) (TPM; Transcripts Per Million).

	Mpc1 (median TPM)	Mpc2 (median TPM)
Heart-Left Ventricle (n = 452)	168.3	50.95
Heart-Atrial Appendage (n = 461)	145.0	44.37
Lung (n = 604)	62.18	28.51
Muscle-Skeletal (n = 818)	74.63	53.09

Supplemental Table 5. Observation of protein interaction using the STRING database from genes that consist of GO:BP (acetyl-CoA biosynthetic process from pyruvate)

#Term ID	Term description	Strength	False Discovery Rate (FDR)	Matching proteins (Interesting targets)
GO:0006850	Mitochondrial pyruvate transmembrane transport	3.44	2.0E-04	Mpc2, Mpc1
GO:0061732	Mitochondrial acetyl-CoA biosynthetic process from pyruvate	3.38	1.7E-18	Pdhx, Pdhb, Mpc2, Pdha1, Dlat, Dld, Mpc1
GO:0006099	Tricarboxylic acid cycle	2.39	7.3E-05	Pdhb, Pdha1, Dlat
GO:1990542	Mitochondrial transmembrane transport	1.98	9.1E-04	Mpc2, Vdac1, Mpc1
GO:0009060	Aerobic respiration	1.81	7.7E-05	Pdhb, Pdha1, Dlat, Dld
GO:0006006	Glucose metabolic process	1.80	2.8E-03	Pdhb, Pdha1, Dlat
GO:0098656	Anion transmembrane transport	1.37	4.9E-02	Mpc2, Vdac1, Mpc1

Supplemental Table 6. KEGG pathways by upregulated genes from the bulk RNA sequencing between CTRL and DOX of 16-week groups

KEGG_PATHWAY (UP)	Term	Count	PValue	Genes
mmu04820	Cytoskeleton in muscle cells	8	1.5E-04	COL3A1, COL5A3, ANKRD1, PKP2, BGN, COL4A5, COL9A2, MYH7
mmu04974	Protein digestion and absorption	6	2.0E-04	COL3A1, COL14A1, COL22A1, COL5A3, COL4A5, COL9A2
mmu04933	AGE-RAGE signaling pathway in diabetic complications	4	1.4E-02	TGFB2, COL3A1, MMP2, COL4A5
mmu04115	p53 signaling pathway	3	5.5E-02	PERP, CYCT, BCL2L1
mmu05410	Hypertrophic cardiomyopathy	3	9.0E-02	TGFB2, ACE, MYH7
mmu05415	Diabetic cardiomyopathy	4	2.0E-02	TGFB2, COL3A1, ACE, MMP2

Supplemental Table 7. Summary of the 10 most downregulated and upregulated differentially expressed genes (DEGs) from cardiac tissue collected from mice 4 weeks after initial exposure to DOX or the control cohort.

Down-regulated	Full name of genes	NCBI Gene Summary
Acr	Acrosin	Acrosin is the major proteinase present in the acrosome of mature spermatozoa
Osgin1	Oxidative Stress Induced Growth Inhibitor 1	This gene encodes an oxidative stress response protein that regulates cell death
Tmem150c	Transmembrane Protein 150C	This gene encodes a transmembrane protein component of a mechanosensitve ion channel that is activated by mechanical stimuli in various cell types and confers slowly adapting, mechanically activated currents in dorsal root ganglion neurons
Scn4b	Sodium Voltage-Gated Channel Beta Subunit 4	The protein encoded by this gene is one of several sodium channel beta subunits
Id1	Inhibitor Of DNA Binding 1	The protein encoded by this gene is a helix-loop-helix (HLH) protein that can form heterodimers with members of the basic HLH family of transcription factors
Ngf	Nerve Growth Factor	This gene is a member of the NGF-beta family and encodes a secreted protein which homodimerizes and is incorporated into a larger complex
Ccl11	C-C Motif Chemokine Ligand 11	This antimicrobial gene is one of several chemokine genes clustered on the q-arm of chromosome 17
Gpcpd1	Glycerophosphocholine Phosphodiesterase 1	Predicted to enable glycerophosphocholine phosphodiesterase activity
Plk2	Polo Like Kinase 2	The protein encoded by this gene is a member of the polo family of serine/threonine protein kinases that have a role in normal cell division

Ctla4	Cytotoxic T-Lymphocyte Associated Protein 4	This gene is a member of the immunoglobulin superfamily and encodes a protein which transmits an inhibitory signal to T cells
**Up-regulated**	**Full name of genes**	**NCBI Gene Summary**
Sprr1a	Small Proline Rich Protein 1A	Gene Ontology (GO) annotations related to this gene include structural molecule activity and protein-macromolecule adaptor activity
Myh7	Myosin Heavy Chain 7	This gene encodes the beta (or slow) heavy chain subunit of cardiac myosin
Adamtsl2	ADAMTS Like 2	This gene encodes a member of the ADAMTS (a disintegrin and metalloproteinase with thrombospondin motifs) and ADAMTS-like protein family
Slitrk4	SLIT And NTRK Like Family Member 4	This gene encodes a transmembrane protein belonging to the the SLITRK family
Mthfd2	Methylenetetrahydrofolate Dehydrogenase (NADP+ Dependent) 2, Methenyltetrahydrofolate Cyclohydrolase	This gene encodes a nuclear-encoded mitochondrial bifunctional enzyme with methylenetetrahydrofolate dehydrogenase and methenyltetrahydrofolate cyclohydrolase activities
Scd4	Stearoyl-coenzyme A desaturase 4	Enables palmitoyl-CoA 9-desaturase activity and stearoyl-CoA 9-desaturase activity
Tgfb2	Transforming Growth Factor Beta 2	This gene encodes a secreted ligand of the TGF-beta (transforming growth factor-beta) superfamily of proteins
Nmrk2	Nicotinamide Riboside Kinase 2	Enables ribosylnicotinamide kinase activity and ribosylnicotinate kinase activity
AI593442	Expressed sequence AI593442	Predicted to be located in membrane. Is expressed in ear; nervous system; and urinary system
Fkbp5	FKBP Prolyl Isomerase 5	The protein encoded by this gene is a member of the immunophilin protein

		family, which play a role in immunoregulation and basic cellular processes involving protein folding and trafficking

Supplemental Table 8. Summary of target and housekeeping proteins for Western blot

Antibody name	Abbreviation	Dilution	Catalog #	Company
❖ Primary				
MPC1 (D2L9I) Rabbit mAb	MPC1	1:1000	14462	Cell Signaling Technology, USA
MPC2 (D4I7G) Rabbit mAb	MPC2	1:1000	46141	Cell Signaling Technology, USA
MCT1 Monoclonal Antibody (P14612)	MCT1	1:500	MA5-18288	Thermo Fisher, USA
MCT4 SLC16A3 Antibody	MCT4	1:500	A05510	Boster Biological Technology
LDHA (C4B5) Rabbit mAb	LDHA	1:1000	3582S	Cell Signaling Technology, USA
LDHB Polyclonal antibody	LDHB	1:1000	14824-1-AP	Proteintech, USA
GPT (E-3), glutamate pyruvate transaminases GPT, also designated alanine aminotransferases (ALT)	ALT	1:500	sc-374501	Santa Cruz, USA
PDH Antibody Cocktail	PDH complex	1:500	45-6799	ThermoFisher, USA
p53	p53	1:500	sc-393031	Santa Cruz, USA
p21	p21	1:500	sc-6246	Santa Cruz, USA
Heat shock protein family D member 1	HSP60	1:1000	12165	Cell Signaling Technology, USA
Vinculin	Vinculin	1:1000	4650	Cell Signaling Technology, USA
❖ Secondary				
Anti-mouse IgG, HRP-linked Antibody	Anti-mouse	1:2000	7076	Cell Signaling Technology, USA
Anti-rabbit IgG, HRP-linked Antibody	Anti-rabbit	1:2000	7074	Cell Signaling Technology, USA

Supplemental Table 9. Body weights of control (CTRL) and doxorubicin (DOX) mice.

Group 1

	**Control (CTRL)**		**Doxorubicin (DOX)**	
**Weeks (W)**	**Number (n)**	**AVER ± SD (g)**	**Number (n)**	**AVER ± SD (g)**
**0**	12	25.5 ± 1.4	30	25.2 ± 1.4
**1**	12	26.3 ± 1.5	30	23.1 ± 1.6
**2**	12	27.0 ± 1.4	30	20.4 ± 2.1
**4**	12	27.5 ± 1.4	27	20.5 ± 2.3
**16**	12	31.9 ± 2.2	19	21.4 ± 3.0

Group 2 (For the PET imaging study)

	**Control (CTRL)**		**Doxorubicin (DOX)**	
**Weeks (W)**	**Number (n)**	**AVER ± SD (g)**	**Number (n)**	**AVER ± SD (g)**
**4**	10	28.2 ± 1.8	10	21.5 ± 0.9

Supplementary Data 1. Time-activity curves and non-linear regressions for [3-^{11}C]pyruvate PET data. Images were processed using the Carimas software package. Non-linear regressions were performed for the 30 min acquisition and the interval between 2 min and 10 min post injection using GraphPad. The file reports the time-activity curves (tab 1), the decay constants of the fitted curves (tab 2), and the transit times (tab 3).

Supplementary Data 2. Uncropped Western blots from which the manuscript figures were constructed.

Figure 1e.

L = protein size ladder

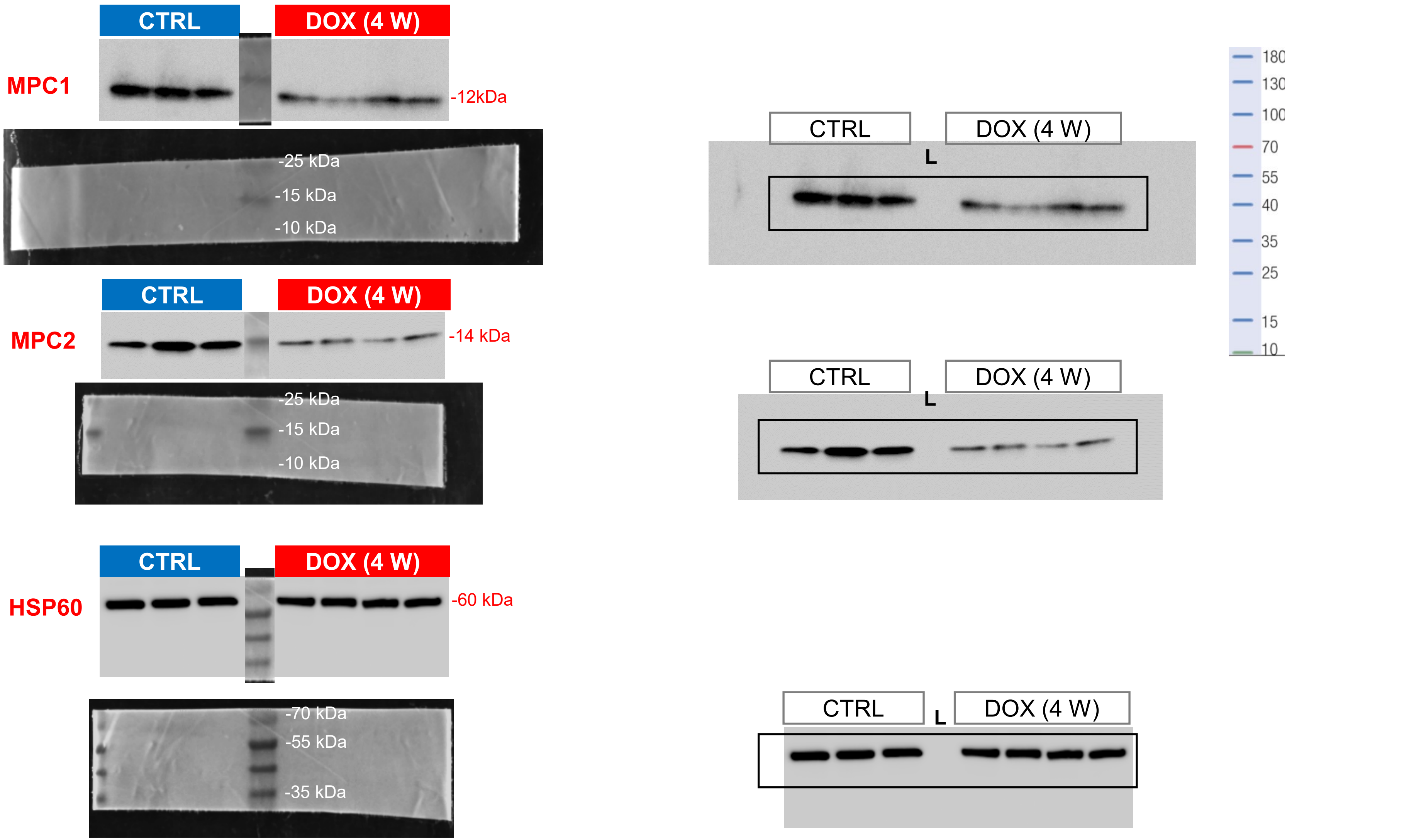

Figure 2b.

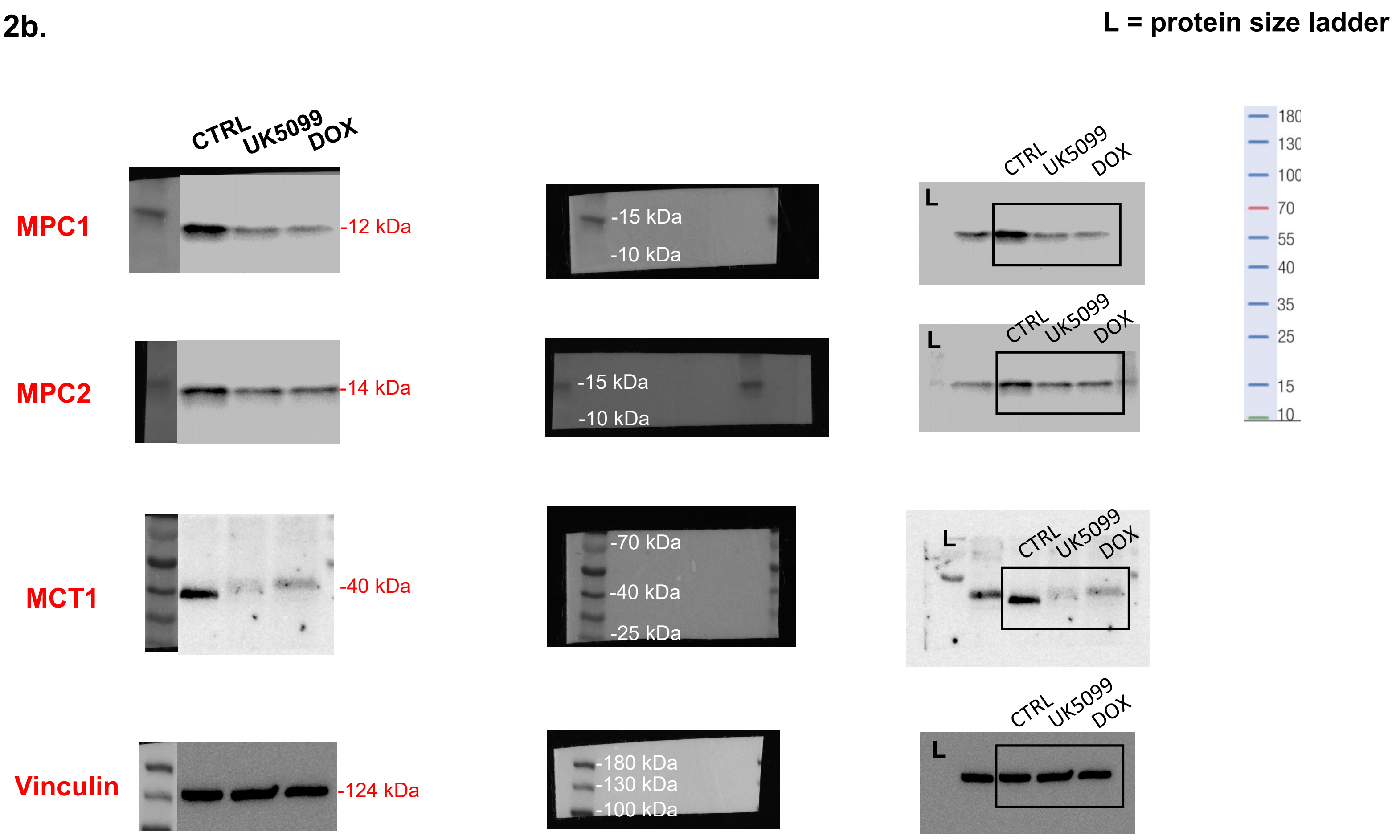
L = protein size ladder
CTRL
UK5099
DOX
MPC1
-12 kDa
MPC2
-14 kDa
MCT1
-40 kDa
Vinculin
-124 kDa
-15 kDa
-10 kDa
-15 kDa
-10 kDa
-70 kDa
-40 kDa
-25 kDa
-180 kDa
-130 kDa
-100 kDa
L
180
130
100
70
55
40
35
25
15
10

Figure 4c.

L = protein size ladder

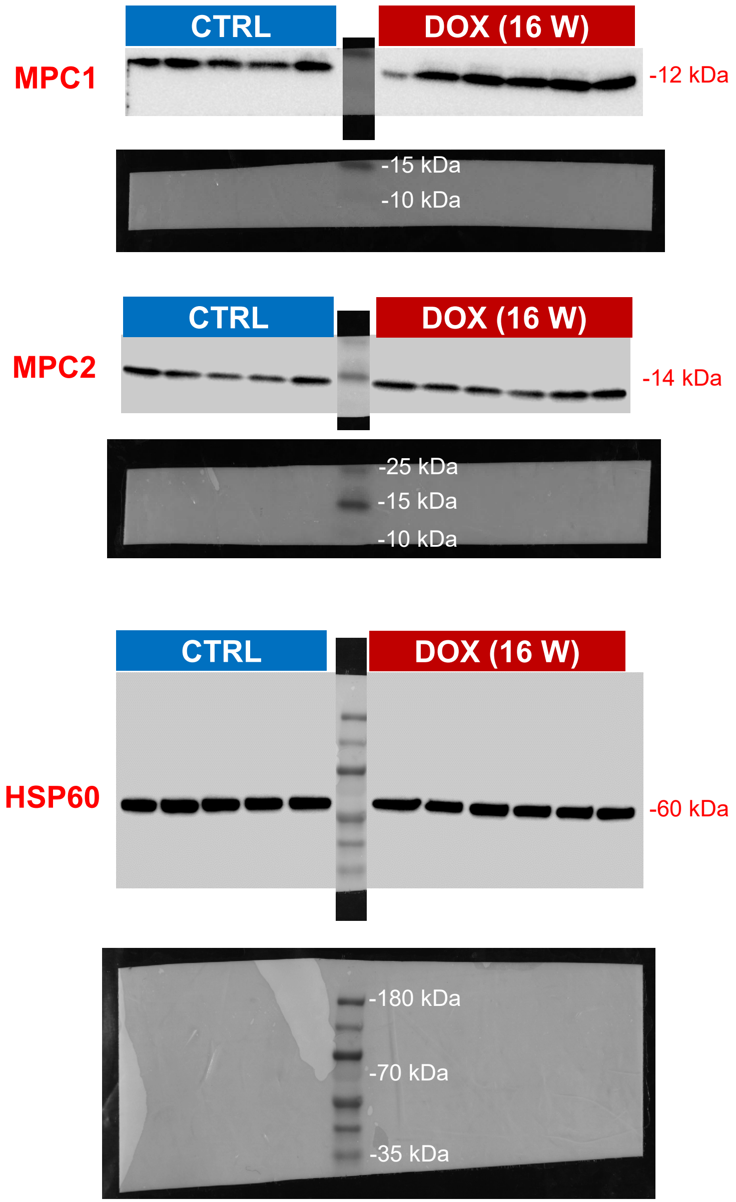

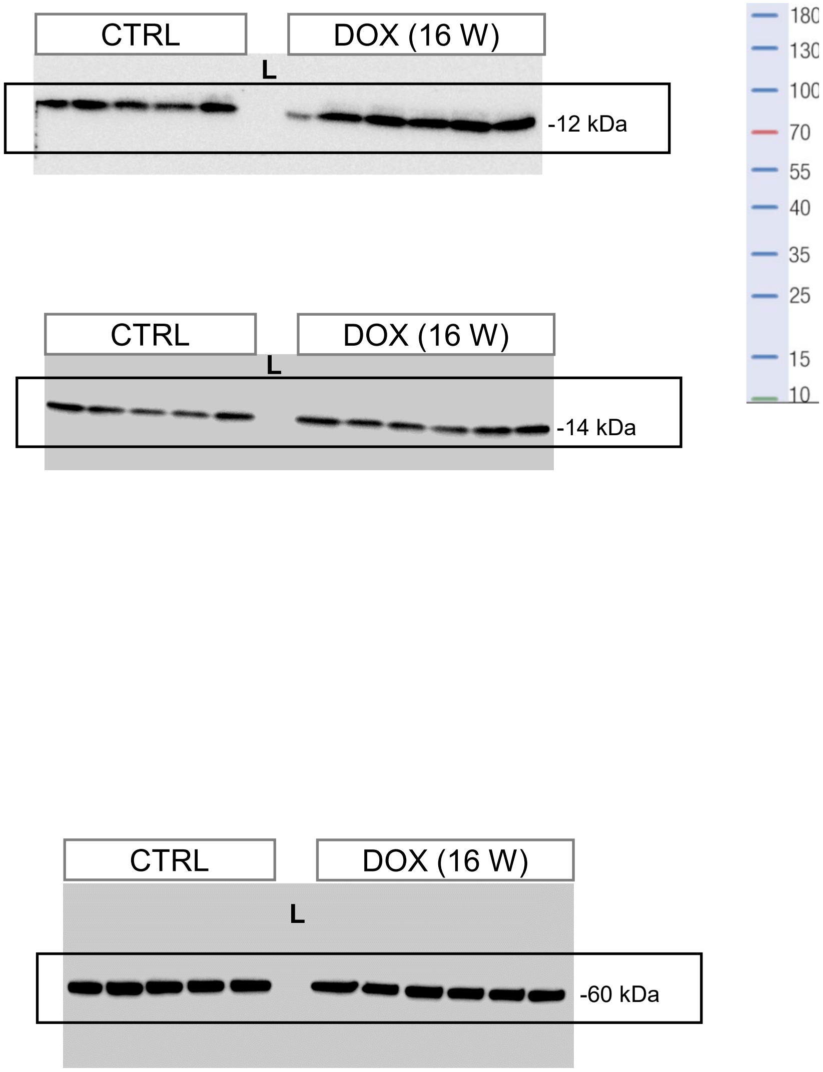

Supplementary Fig. 1e

L = protein size ladder

*The theoretical molecular weight of **MCT1** is approximately 54 kDa, which was also confirmed in our <u>tissue-derived samples</u>, showing a band around 55 kDa. However, since the antibody used in this study is reported by the manufacturer to detect MCT1 at approximately 40 kDa, we accordingly indicated 40 kDa in the figures.*

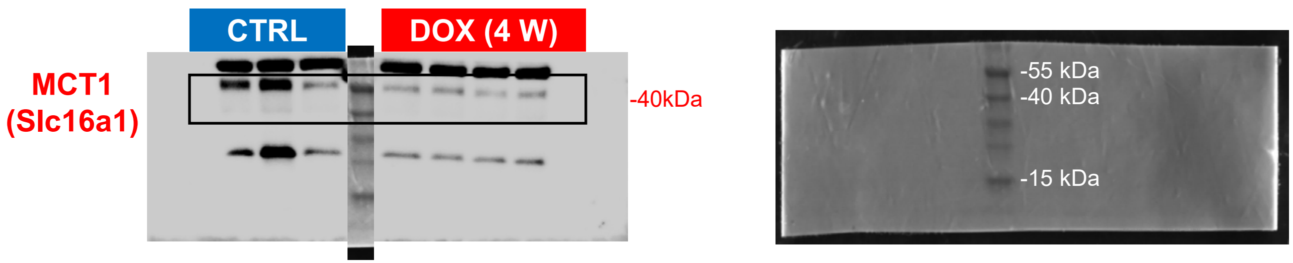

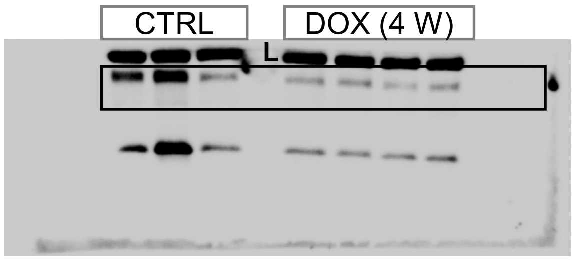

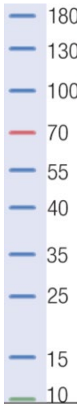

MCT1's internal control, HSP60, is same in Figure 1d

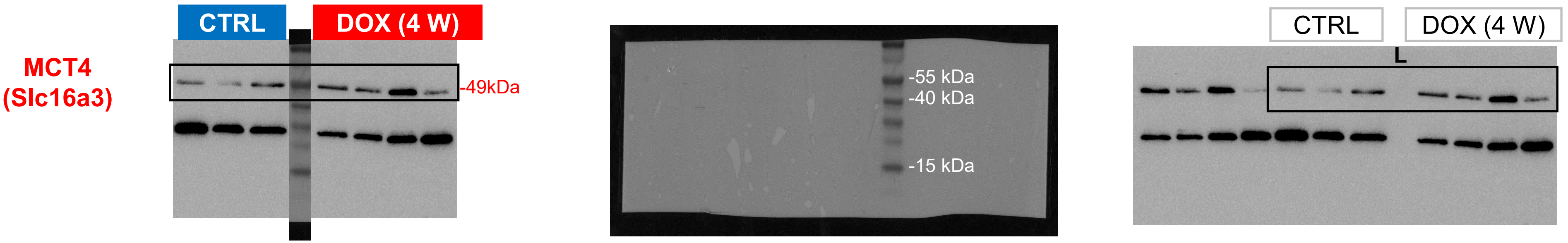

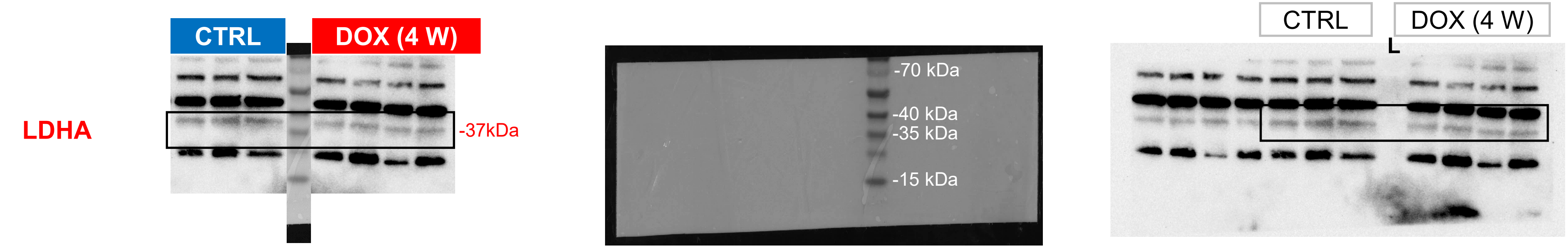

Supplementary Fig. 1e (continue)

L = protein size ladder

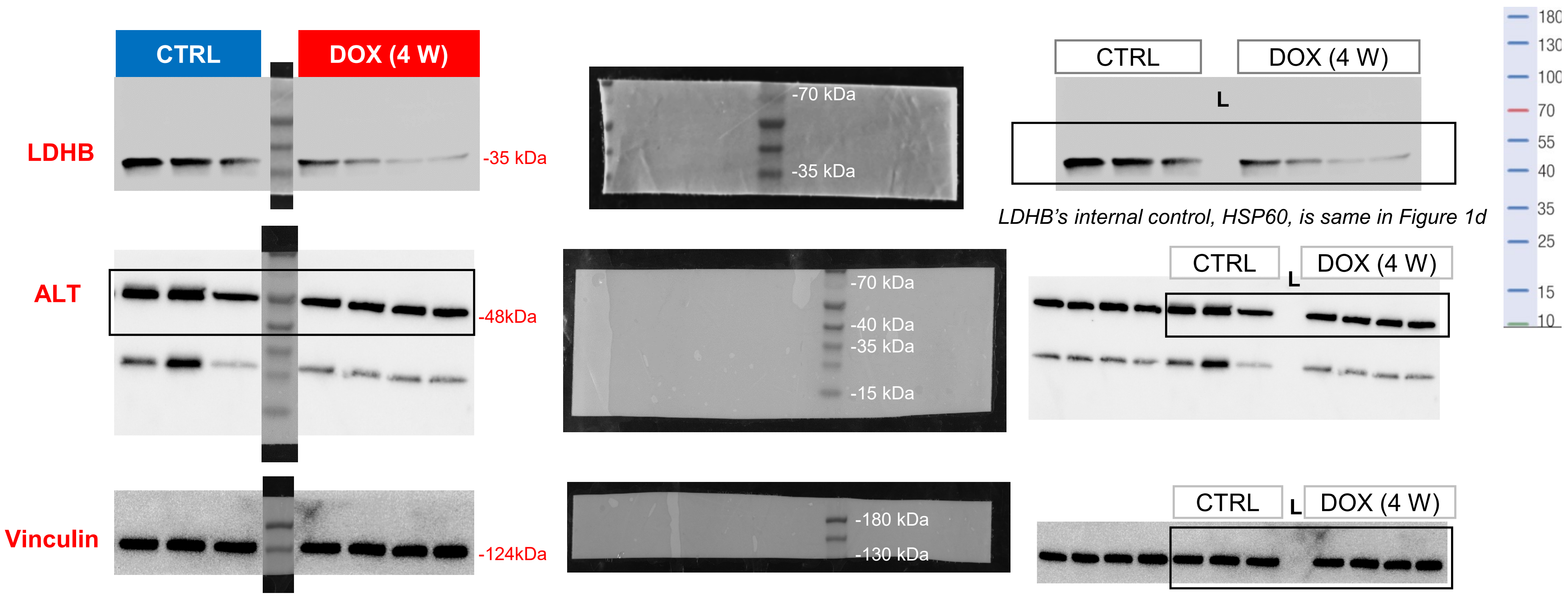

Supplementary Fig. 2e

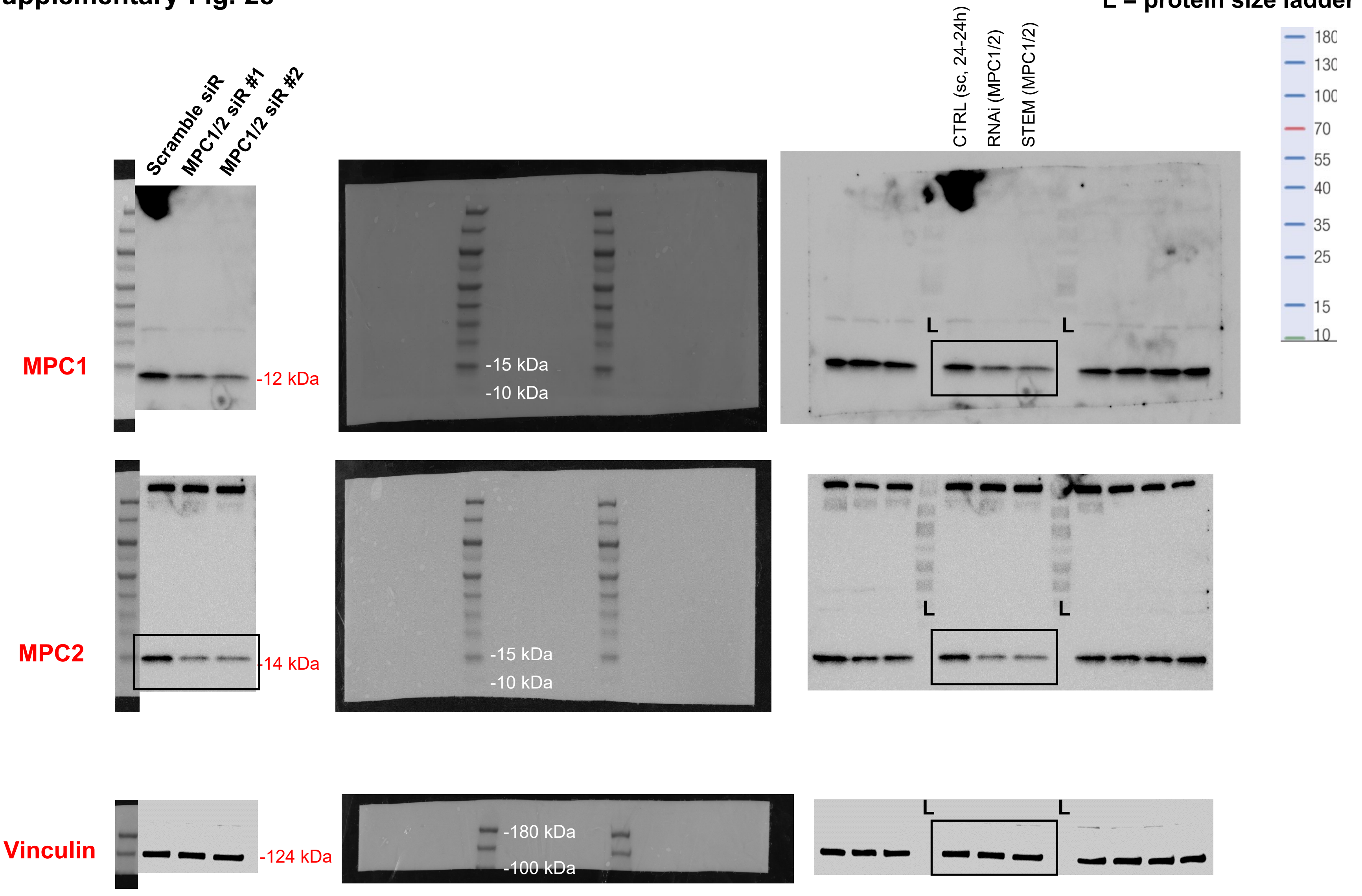

Supplementary Fig. 2f

L = protein size ladder

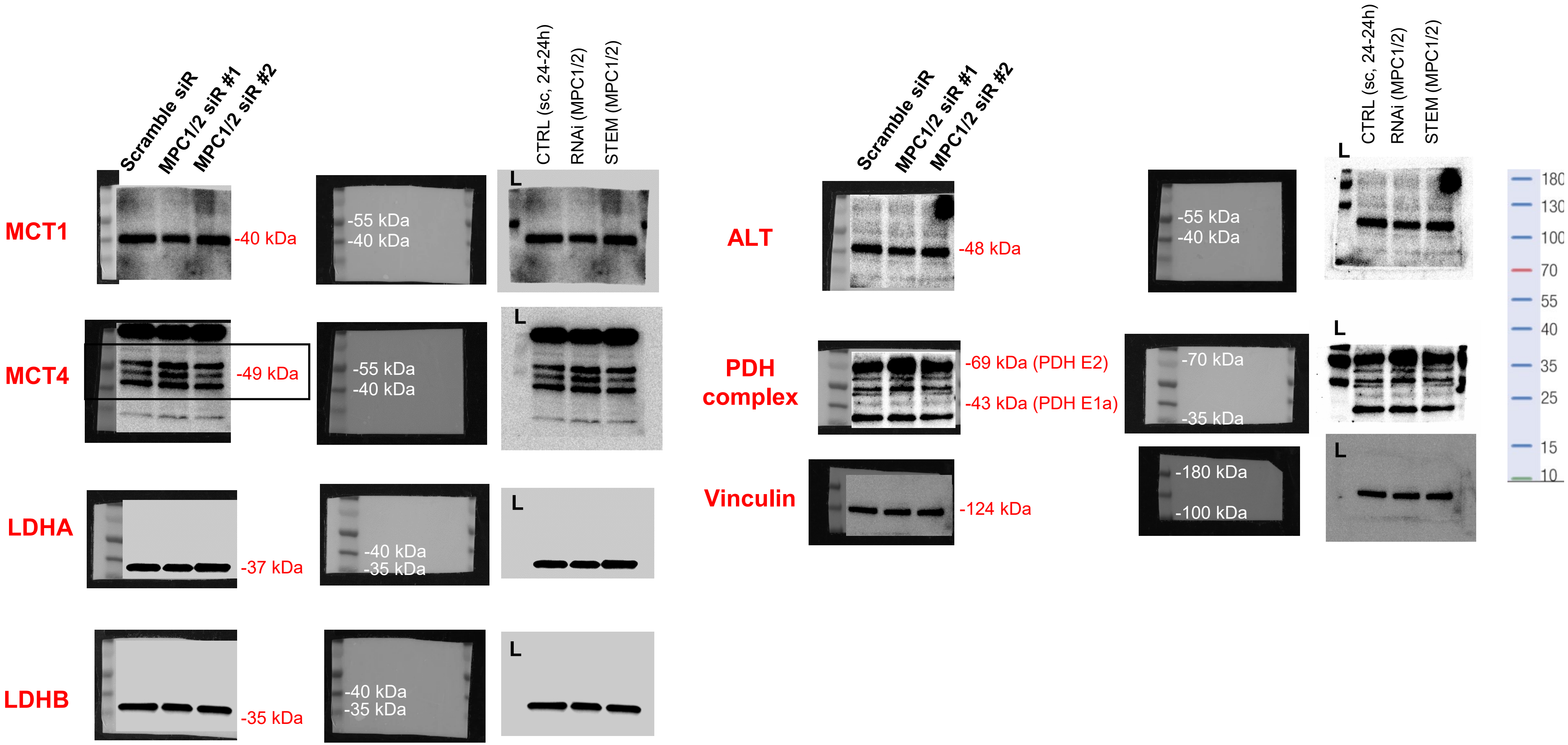

Supplementary Fig. 4b

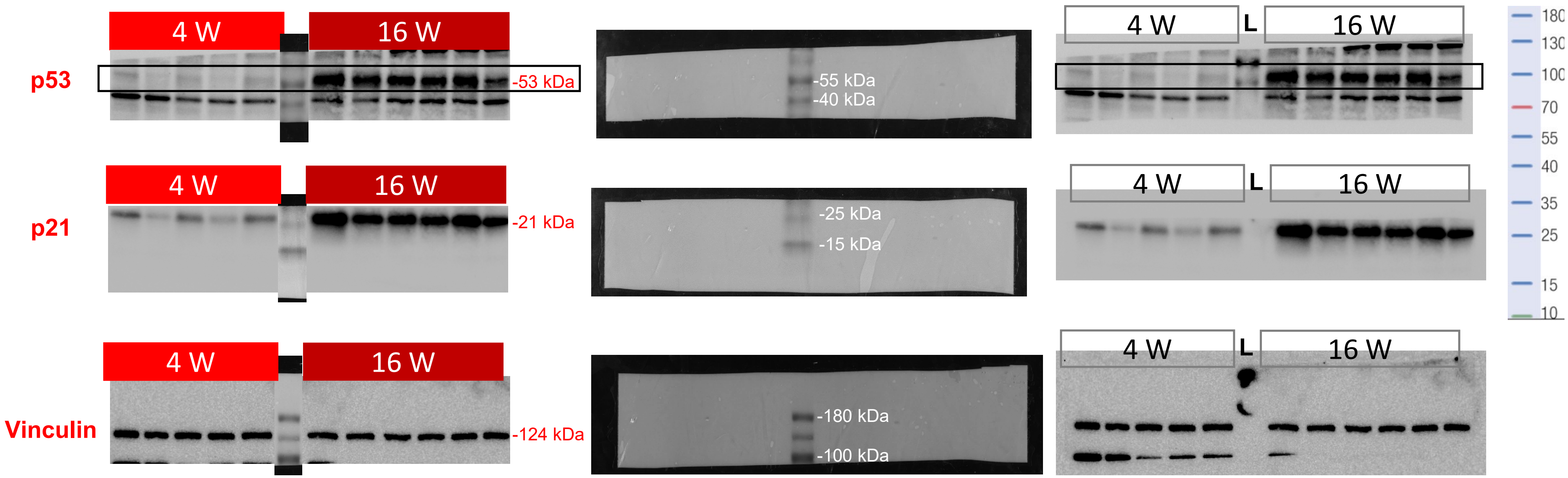

Supplementary Fig. 4d

L = protein size ladder

*The theoretical molecular weight of **MCT1** is approximately 54 kDa, which was also confirmed in our <u>tissue-derived samples</u>, showing a band around 55 kDa. However, since the antibody used in this study is reported by the manufacturer to detect MCT1 at approximately 40 kDa, we accordingly indicated 40 kDa in the figures.*

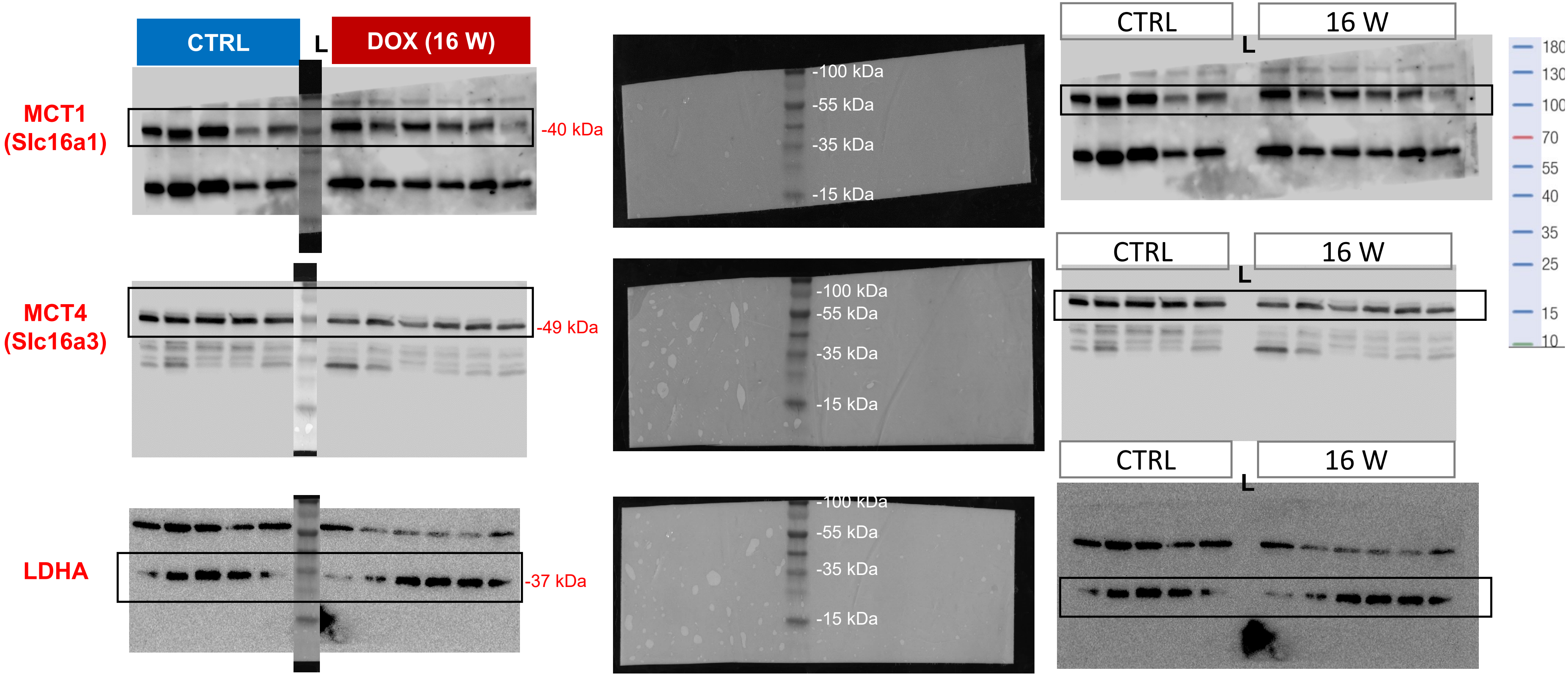

Supplementary Fig. 4d (continue)

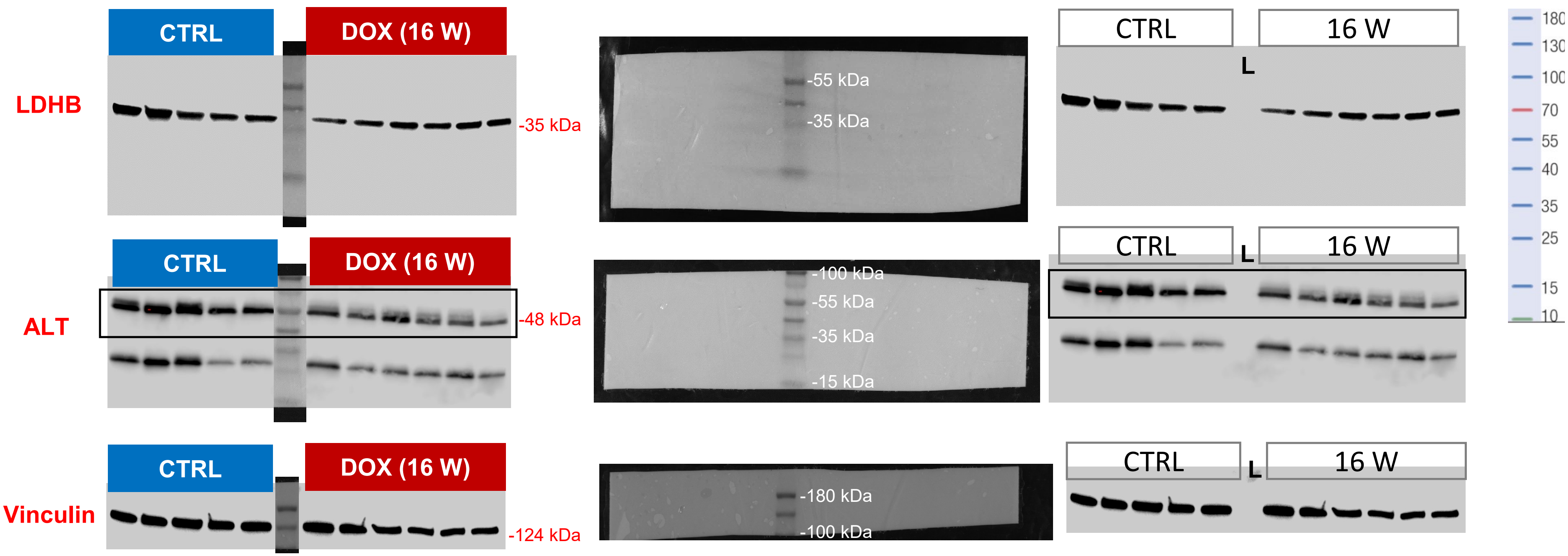

Supplement: Supplementary file 1 — Supplementary information [file 44303_2026_165_MOESM1_ESM.pdf]
